# The hydrogen puzzle in rock-enhanced biochar: Pyrogenic coating, mineral redox and pore accessibility

**DOI:** 10.1371/journal.pone.0355781

**Published:** 2026-08-12

**Authors:** Johannes Meyer zu Drewer, Tobias Bromm, Thomas D. Bucheli, Pellegrino Conte, José María de la Rosa, Harald Fitzek, Amin Ghafarpour, Bruno Glaser, Andreas Kappler, Calogero Librici, Jens Möllmer, Michael Oberaigner, Sara Maria Pérez-Dalí, Arka Rudra, Águeda Sánchez-Martín, Hamed Sanei, Hans-Peter Schmidt, Nikolas Hagemann

**Affiliations:** 1 Ithaka Institute, Goldbach, Germany; 2 Environmental Analytics, Agroscope, Zürich, Switzerland; 3 Institute of Sustainable Energy Systems, Offenburg University, Offenburg, Germany; 4 Institute of Agricultural and Nutritional Sciences, Martin Luther University Halle-Wittenberg, Halle, Germany; 5 Department of Agricultural, Food and Forest Sciences, University of Palermo, Palermo, Italy; 6 Instituto de Recursos Naturales y Agrobiología de Sevilla, IRNAS-CSIC, Seville, Spain; 7 Graz Centre for Electron Microscopy (ZFE), Graz, Austria; 8 Institute of Electron Microscopy and Nanoanalysis (FELMI), Graz University of Technology, Graz, Austria; 9 Terrestrial Paleoclimatology and Paleontology, Eberhard Karls University Tübingen, Tübingen, Germany; 10 Geomicrobiology, Department of Geosciences, University of Tuebingen, Tuebingen, Germany; 11 Cluster of Excellence: EXC 3121: TERRA – Terrestrial Geo-Biosphere Interactions in a Changing World, University of Tübingen, Germany; 12 Cluster of Excellence EXC 2124, Controlling Microbes to Fight Infection, University of Tuebingen, Tuebingen, Germany; 13 Institute for Non-Classical Chemistry e.V. (INC), Leipzig, Germany; 14 Lithospheric Organic Carbon Group, Department of Geoscience, Aarhus University, Aarhus, Denmark; 15 Ithaka Institute, Arbaz, Switzerland; National Chung Cheng University College of Engineering, TAIWAN

## Abstract

Rock-enhanced (RE-)biochars, produced by co-pyrolysis of silicate rock powder with biomass, were proposed to combine pyrogenic carbon capture and storage (PyCCS) with enhanced rock weathering (ERW) as complementary carbon dioxide removal approaches. Catalytic effects of rock-derived AAEM on pyrolysis and carbon speciation were anticipated. Here we produced six RE-biochars (10–90% rock content) by co-pyrolysis of basanite rock powder with wood at contrasting highest treatment temperatures of 450 °C and 750 °C. To provide an in-depth physico-chemical characterization of the RE-biochars, focusing on the biochar-mineral interface region, analytical methods including random reflectance, fast field cycling proton nuclear magnetic resonance relaxometry and electron energy loss spectroscopy were employed. While most treatments had limited effect on the biochar carbon yield (y_c_) and aromaticity of the pyrogenic carbon, RE-biochar produced at 750 °C and containing 90% rock showed an increased hydrogen to carbon molar ratio (H:C_org_), which we partly explained by i) contribution of geogenic hydrogen, ii) physical adsorption of hydrogen to minerals and iii) pyrogenic coating of rock particles with secondary char containing aliphatic- and carboxylic moieties. Still, we cannot fully identify the reasons for increased hydrogen content with constant aromaticity. A decreased reflectance (Ro) and thermal stability (BC_1000C_) for RE-biochars with 90% rock content, despite similar aromaticity as quantified by hydropyrolysis, highlight the limitations of analytical methods developed for carbonaceous materials in the assessment of high ash biochars. Co-pyrolysis can reduce metal oxides, such as hematite (Fe-III) to magnetite, producing (potentially) reactive metals species and can accumulate geogenic potassium (K) at micron scale at rock particle surfaces. This study provides evidence that co-pyrolysis of biomass and basanite does not increase y_c_ but alters the mineral speciation and elemental distribution at the surface of rock particles. These findings may be further exploited in the design of functionalized RE-biochars and in ERW research.

## 1. Introduction

The co-pyrolysis of biomass with exogeneous minerals was suggested to design functional biochars [1,2]. The presence of mineral matter in biomass pyrolysis impacts the relative yields of gaseous, liquid, and solid pyrolysis products as well as their carbon speciation. Those effects were shown for minerals inherently present in biomass [3] and for additives like salts, or wood ash [4–6]. The deliberate addition of mineral matter to the feedstock before pyrolysis is referred to as co-pyrolysis. Potassium salts can, e.g., improve phosphorus availability in sewage sludge char by the formation of soluble potassium hydrogen phosphate during pyrolysis [7]. Silica, iron salts and other metals were applied to improve the capacity of biochar to sorb organic micropollutants [8]. Underlying mechanisms may include alterations to biochar carbon speciation and porosity, but also the formation of specific mineral surfaces during pyrolysis, e.g., the reductive formation of magnetite, a mixed valence iron mineral from the Fe(III) mineral siderite [9] or the formation of Fe(0) from Fe(III)-hydroxide [10].

Mineral additives can also increase the biochar carbon yield (y_c_), i.e., the fraction of biomass carbon converted to solid state pyrogenic carbon (PyC) during (co-)pyrolysis [11–13]. This was demonstrated for biomass co-pyrolyzed with wood-ash [6] or potassium acetate [5]. Improved mass conversion is mediated by catalytic effects [12], the formation of organo-metallic bonds [14], or the (partial) encapsulation of biomass [8,15]. Alkali and alkaline earth metals (AAEM) contained in mineral additives appear to be most relevant for the catalytic reduction of activation energy for biomass decomposition and the promotion of C–C cross-linkage formation, which together increases secondary char formation [6,16].

Silicate rock powder, as employed for enhanced rock weathering (ERW) [17–19] and for soil remineralization [20–22], was suggested as an abundant mineral additive to produce rock-enhanced (RE-)biochar [23–25]. Meyer zu Drewer *et al.* [24] showed that rock powder particles received a pyrogenic coating during co-pyrolysis, i.e., secondary char condensates on their surfaces, which could enhance y_c_ by increasing secondary char formation. However, y_c_ and aromaticity remained unaltered despite rock powder addition. Alkali and alkaline earth metals, when embedded in a (crystalline) silicate rock matrix, seem to remain, opposite to the above, largely unexposed and non-reactive at highest treatment temperatures (HTT) of 650 °C. Since rock powder has a significantly higher thermal conductivity than biomass, it has been suggested that this leads to a higher pyrolysis intensity (i.e., higher heating rate and thus in longer exposure to the HTT) within biomass-rock powder pellets compared to pure biomass [22].

In the present study, we investigate how co-pyrolysis is altering the physico-chemical properties of RE-biochar, compared to pure biochar. We expand previous characterisations of RE-biochars [24,25] with a novel set of samples and a focus on microsites at the biochar-mineral interface, where a distinct PyC and mineral speciation may form during pyrolysis. Therefore, we produced six RE-biochars by co-pyrolysis of softwood with basanite rock powder at 450 °C and 750 °C. The RE-biochars contained 10 wt% (9:1), 50 wt% (1:1), or 90 wt% (1:9) basanite rock powder alongside two pure softwood biochars (BC450, BC750). Using random reflectance (Ro), hydropyrolysis (HyPy), benzene polycarboxylic acids (BPCA) molecular marker method, fast field cycling relaxometry proton nuclear magnetic resonance spectroscopy (FFC ^1^H-NMR) and scanning transmission electron microscopy coupled to electron energy loss spectroscopy (STEM-EELS) and other analytical methods, we characterized the PyC speciation and discussed potential mechanisms affecting the latter. Both weathering and alkalinity production as well as the effect on plant growth of the (RE-)biochars characterized here, were investigated in Meyer zu Drewer et al. 2026 [26].

## 2. Results and discussion

### 2.1. Increased H:C_org_ ratio despite similar aromaticity: Where does the hydrogen come from?

Biochars were produced from pelleted, low-ash wood shavings (biomass composition S1 Table) at 450 °C and 750 °C in a continuously operated auger reactor [27]. These showed well-expected properties, mass yield (y_m_), and y_c_ as shown in Table 1 [28–30]. Based on the y_m_ of the pure biochars, we mixed basanite rock powder (elemental and mineral composition, S2 Table) to the wood shavings prior to pelleting and pyrolysis to achieve the desired rock powder content in the RE-biochar.

**Table 1:** Basic physico-chemical properties of biochar and rock-enhanced biochar (BC) produced at 450 °C (450) or 750 °C (750), containing 0 (-), 10% (9:1), 50% (1:1) or 90% (1:9) basanite rock powder. Data on main- and trace element content are presented in S7 Table and basic properties of basanite in S2 Table).

	BC450	BC450–9:1	BC450–1:1	BC450–1:9	BC750	BC750–9:1	BC750–1:1	BC750–1:9
**Mass yield (y**_**m**_ **in%)**^**1**^	25.6 ± 0.5	28.0 ± 0.3	40.5 ± 1.2	72.7	18.9 ± 0.7	24.7 ± 0.1	33.4 ± 0.2	66.4
**Carbon yield (y**_**c**_ **in %)**^**1**^	42.9 ± 0.9	43.7 ± 0.4	40.3 ± 1.2	46.6	35.2 ± 1.2	41.3 ± 0.2	35.5 ± 0.2	38.3
**Rock content (%)**	0	5.7	50.2	89.1	0	8.3	49.9	89.1
**Biogenic content (%)**	100	94.3	49.8	10.9	100	91.7	50.1	10.9
**Total ash (%)**	1.9	7.5	51.2	89.3	3.0	11.0	51.3	89.4
**Biogenic ash (%)**	1.9	1.8	1.0	0.2	3.0	2.7	1.4	0.3
**Carbon (C, %)**	85.0	76.7	41.5	9.3	94.3	83.6	46.2	9.7
**Organic C (C**_**org**_ **,%)**	84.8	76.7	41.5	9.3	93.9	83.2	45.8	9.6
**C**_**org_daf**_ **(% of C**_**org**_)	86.4	87.1	86.1	86.1	96.5	95.0	96.0	98.0
**C**_**vol.HAWK**_ **(mg g**^**-1**^ **C**_**org**_**)**^*2*^	10.9	11.7	10.7	12.9	0.3	0.4	0.7	3.3
**BC**_**HyPy**_ **(% of C**_**org**_)^**3**^	56.4	58.4	49.5	42.7	97.3	95.6	97.4	97.2
**TIC (%)** ^**4**^	0.2	< 0.1	< 0.1	< 0.1	0.4	0.4	0.4	0.1
**Hydrogen (H, %)**	3	2.9	1.7	0.4	1.3	0.8	0.5	0.7
**Nitrogen (%)**	0.29	0.06	< 0.05	< 0.05	0.33	0.41	0.15	< 0.05
**Sulfur (%)**	< 0.03	< 0.03	< 0.03	< 0.03	< 0.03	< 0.03	< 0.03	< 0.03
**Oxygen (%)**	9.8	8.4	5	1.2	1.3	2.8	0.9	<LOQ
**H:C**_**org**_ **molar ratio**	0.42	0.45	0.49	0.51	0.16	0.11	0.13	0.87
**H**_**Bio**_**:C**_**org**_ **molar ratio**^**5**^	0.42	0.45	0.44	0.17	0.16	0.11	0.09	0.54
**SEC (µS cm**^**-1**^)^**6**^	6.0x10^-5^	4.7 x10^-5^	1.6 x10^-5^	<9.6x10^-7^	1.3 x10^3^	1.7 x10^3^	7.6 x10^2^	4.5 x10^1^
**SSA (m**^**2**^ **g**^**-1**^)^**7**^	1.4	4.3	3.3	9.1	447	421	231	23
***∑BPCA-3 (%)*** ^***8***^	7.6	7.8	8.5	8.0	0.9	0.8	1.0	1.4
***∑BPCA-4 (%)***	26.5	27.3	27.0	28.4	6.4	6.0	6.0	7.8
***∑BPCA-5 (%)***	38.3	39.5	37.5	40.2	21.7	19.8	30.8	21.2
***∑BPCA-6 (%)***	27.6	25.4	27.1	23.2	71.0	73.4	72.2	69.6
***∑BPCA 3−6 (g C kg*** ^***-1***^ **)**	106.1	86.7	47.3	13.8	125.6	111.1	74.9	13.7

^1^Variability *calculated as mean x coefficient of variation (CV%) of biochar transport-rate (t*_*b*_*; n = 3), not available for BC450–1:9 and BC750–1:9.*

^*2*^ *Fraction of carbon being volatilized between 300–650 °C during analytical pyrolysis on a HAWK-pyrolizer.*

^3^Fraction of organic carbon resistant to hydropyrolysis.

^4^Total inorganic carbon.

^5^*H:C*_*org*_ *molar ratio of the biogenic fraction, i.e., bulk H:C*_*org*_ *corrected for geogenic hydrogen and carbon in the sample (Equation 6).*

^*6*^*Solid state electric conductivity. Limit of detection is sample specific, calculated from biochar pellets height after compression at 160 MPa (BC450–1:9 = 4.5 mm distance between anode and cathode) and maximum resistivity measurable with the Black Gauss I (*100 x 10^6^ Ohm). *LOQ = Limit of quantification. SEC = solid-state electrical conductivity.*

^7^Specific surface area quantified by nitrogen gas adsorption and quantified using BET-method.

^8^*BPCA =* *Benzenepolycarboxylic acid marker as detailed in the Methods.*

Rock addition increased the ash content of the biochar in agreement with the rock amendment rate. Dry and ash-free (daf) organic carbon (C_org_) content and y_c_ remained largely constant for RE-biochars produced at the same HTT (Table 1). The most remarkable effect of rock addition was the extremely high H:C_org_ molar ratio of 0.87 observed for the RE-biochar produced at 750 °C with 90% rock content (BC750–1:9). As pure basanite showed 0.3% hydrogen content in elemental analysis (0% C), we used this data to calculate a H:C_org_ molar ratio of the biogenic fraction, which was still considerably higher than expected (H_bio_:C_org_ = 0.54). This calculation assumes that the rock, or at least its H content, remained unchanged by co-pyrolysis, which we examine in more detail below.

Lower dosages of rock powder had a limited effect on this biochar property and rather decreased the H:C_org_ molar ratio at 750 °C. At 450 °C, H:C_org_ molar ratio increased only slightly with increasing rock content. To verify these results, we reproduced all samples (S3 Table) with the difference that pelleting was not carried out in the lab but by a service provider and thus presumably under different pressure conditions (higher pellet density in 2^nd^ batch). In this 2^nd^ batch, the BC750–1:9 sample had a H:C_org_ molar ratio of 0.67 (H_bio_:C_org_ = 0.37) (S4 Table), which is lower than in the first experiments but still much higher than the control and generally higher than expected for a biochar made at this HTT. Such high H:C_org_ molar ratios are almost unprecedented at a HTT of 750 °C [29,30]. Yang et al. [13] have documented an increase of H:Corg molar ratios in biochar produced at 500 °C from 0.2-0.3 to 0.3-1.3 for the addition of various minerals and salts, including Ca(H_2_PO_4_)_2_, which caused the maximum values.

Still, a quantification of BPCA markers (%BPCA-6) [31] and hydropyrolysis (HyPy) [32,33] did not reveal any considerable changes due to rock enhancement, which strongly suggests that changes in H:C_org_ ratio (Table 1) are not the result of changes in biochar’s aromaticity. For HTT 450 °C, BPCA-6 – the hexacarboxylated benzene marker indicative of highly condensed aromatic domains in biochar, accounted for 23.3%−27.6% of total BCPAs and 42.7%−58.4% of C_org_ resisted HyPy (BC_HyPy_), representing the aromatic carbon moieties of eight or more condensed rings. For HTT 750 °C, BPCA-6 presented 69.6%−73.4% of total BCPAs and BC_HyPy_ 95.6%−97.4% of C_org_. Also, absorption spectra obtained by Fourier-transformed infrared spectroscopy (FTIR) and Raman spectroscopy revealed no difference in bulk carbon speciation between (RE-)biochars produced at the same HTT. Fourier transformed infrared spectroscopy showed the presence of aromatic C = C bonds at 1589 cm^-1^ and absence of symmetric- and asymmetric aliphatic carbon structures (2840-2890 cm^-1^ and 2920−2990 cm^-1^, respectively) in all samples (S5 Fig). Raman spectroscopy revealed that ratios of D/G intensity- and area ratios were indifferent between samples obtained at the same HTT (S6 Fig). The G band at 1600 cm^-1^ relates to C-C linkages in aromatic moieties, i.e., the degree of graphitization. The D band at 1345 cm^-1^ relates to structural defects of the latter [34]. As the aromatic backbone remained largely unchanged compared to the control biochar, the increase in the H:C_org_ molar ratio can only be explained by an increase of the H content of the mineral phase and/or an increase of the H:C_org_ molar ratio in the non-BC_HyPy_ fraction. This may result from aliphatic compounds (e.g., alkanes, H:C ≈ 2) in the pyrogenic coating of the rock surface [24]. This aliphatic- or small aromatic systems would be included in measured non-BC_HyPy_ fraction or the S1&S2 fraction of sequential HAWK pyrolysis (c.f. section 2.1.1).

#### 2.1.1. Hydrogen bound in semi-volatile organic compounds.

**Fig 1:**
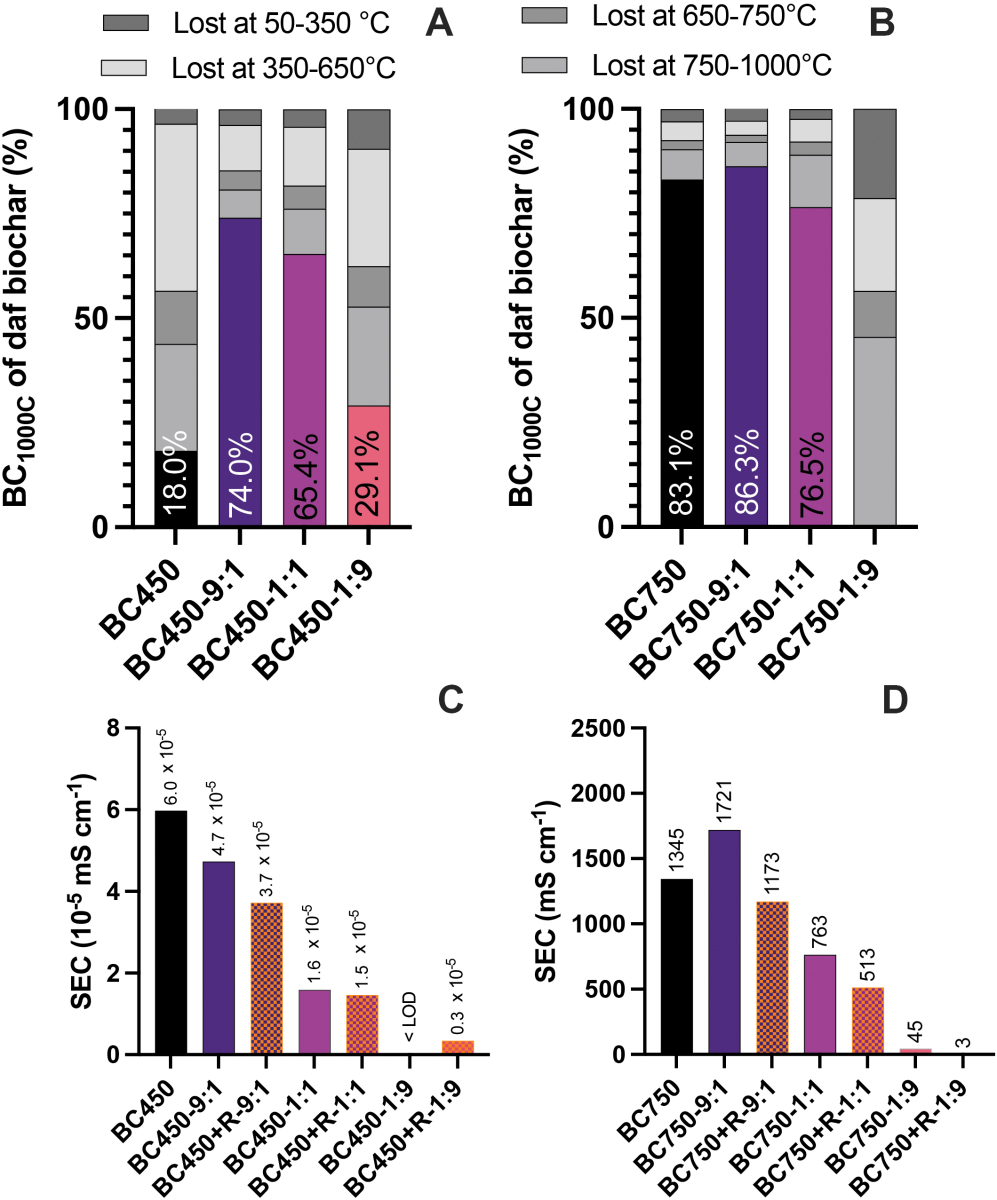
Top: Thermogravimetric analysis of rock-enhanced biochars: Mass loss (%) of dry and ash free (daf) biochar in specific temperature ranges (in gray scale) and fraction retained at 1000 °C (BC_1000C_). Bottom: *Solid-state electrical conductivity (SEC) in mS cm*^-1^. (A + C): *Rock-enhanced biochars pyrolyzed at 450 °C (BC450) containing 10-90% basanite (BC450-9:1, BC450-1:1 and BC450-1:9),* (B + D): *Rock-enhanced biochars pyrolyzed at 750 °C (BC750), containing 10-90% basanite (BC750-9:1, BC750-1:1 and BC750-1:9). Orange shaded bars with BC + R notation denote equivalent post pyrolysis mixtures of biochar and rock powder. LOD = Sample specific limit of detection, calculated from biochar pellets height after compression at 160 MPa (4.5-6.0 mm = distance between anode and cathode) and maximum resistivity measurable with the setup (*100 x 10^6^ Ohm).

An increase in non-BC_HyPy_ should correspond to a decrease in thermal stability and an increase in thermally labile, i.e., volatile organic compounds. We quantified the daf mass loss (m_vol,TGA_) of 5 mg (RE-)biochar in the range of 50−1000 °C at 10 °C min^-1^ thermo-gravimetric analysis (TGA). In parallel, a HAWK-pyrolizer with coupled C_org_-analyzer quantified carbon loss (C_vol,HAWK_) from 10 mg sample in the range of 300−650 °C at 25 °C min^-1^ [35]. Both analyses ran under inert atmosphere. The content of C_vol,HAWK_ (Table 1) increased with rock powder content from 10.9 and 0.3 mg C_vol,HAWK_ per gC_org_ at 0% rock to 12.9 and 3.3 mg C_vol,HAWK_ per g^-^C_org_ for biochars containing 90% rock that were produced at HTT of 450 °C and 750 °C, respectively. The increase was small at 10% and 50% rock but pronounced at 90% rock content. Likewise, m_vol,TGA50−350°C_ was 2−4% of daf biochar for samples with 0−50% rock content but 9% from BC450–1:9 and 21% from BC750–1:9 (Fig 1). The same trend is observed for m_vol,TGA350−650°C_ (corresponding to the HTT of the HAWK pyrolizer), except for BC450. Still, the C_vol,HAWK_ fraction of daf biochar is < 1% and cannot solely explain the reduced thermal stability observed in equivalent temperature ranges. The fraction of daf biochar ultimately resisting to thermal degradation at the end of the TGA temperature ramp is referred to as BC_1000C_ (%). The strongly increased BC_1000C_ fraction in RE-biochars produced at HTT 450 °C, same as the complete loss of BC_1000C_ from the BC750–1:9 sample cannot rationally be explained by their initial PyC speciation (Fig 1). We elucidate potential limitations of methods, which were initially developed for carbonaceous materials, in the thermal analysis of RE-biochars in section 2.5.

The solid-state electrical conductivity (SEC) of RE-biochar decreased compared to pure biochar, which was expected because silicate rock is an electric insulator. However, SEC was higher than in the equivalent post-pyrolysis mixtures (PPMs) of pure biochar and rock powder (Fig 1C + D). These results are consistent with Meyer zu Drewer *et al.* [24], who co-pyrolyzed the same basanite with landscaping wood on the same pyrolysis unit at 650 °C. They attributed the increased SEC to pyrogenic coatings of rock particles with conductive, soot-like secondary char formed from gaseous pyrolysis products [36,37]. Such secondary char contains condensed aromatic structures and condensates of tar [38]. Condensates are typically more hydrogenated than a primary aromatic lattice [39], which may partially explain the elevated H:C_org_ molar ratio of RE-biochars. Sorption of volatile organic carbon onto SiO_2_-rich mineral surfaces [40] can provide nucleation sites for secondary char. Both in Meyer zu Drewer *et al.* [24] and in the present study, rock enhancement did not change y_c_. Thus, the amount of carbon from secondary char coating is quantitatively minor compared to the total carbon yield. It can be concluded that small, hydrogenated carbon species, as approximated by C_vol.HAWK_ content (dummy H:C_org_ ratio = 2; aliphatic carbon) and non-BC_HyPy_ content (dummy H:C_org_ ratio = 0.625; pyrene) contribute only to a negligible extend to the total H content of the biochar (S8 Table).

#### 2.1.2. Hydrogen associating or reacting with minerals.

Gaseous hydrogen (H_2_) is an inherent by-product of biomass pyrolysis [41–43]. Its production increases with HTT and may exceed 50 vol% of the pyrolysis gas depending on feedstock selection and process conditions [44,45]. Physi- and chemisorption of H_2_ to silicate minerals can occur in consequence of a solid-gas exchange where H_2_ or other H-bearing volatiles are present. The sorption of H_2_ to minerals is dominated by physical adsorption [46]. Silicate minerals can be polar due to isomorphic substitution, charged surfaces and edges or functional groups such as silane groups (Si-OH). Hydrogen can easily be polarized and bind to charged mineral surfaces by van der Waals forces [47]. The adsorption capacity increases with micro porosity and specific surface area (SSA) [46]. Consequently, H_2_ sorption to dense minerals such as pyroxene or plagioclase is likely limited but considerable for amorphous Fe-oxides and layered silicates. Especially defect sites and the interlayer space (>2.9 Å) in 2:1-layer silicates are relevant for H_2_ adsorption [43–45,48]. Catalytic metals can further enable hydrogen spillover to surfaces that otherwise do not bind H under identical conditions [49].

Lastly, H (respectively H_2_O) can also be structurally incorporated in nominally anhydrous minerals and isomorphically substitute geogenic metal ions such as Mg^2+^. This was demonstrated for olivine under extremely high pressure and temperature regimes. At 1 atm, this process is much more limited and likely restricted to surface sites. Natural, nominally anhydrous olivine contains < 0.05 mol H kg^-1^ [50,51]. Sundvall & Skogby (2011) [52] investigated the protonation of pyroxene under varying conditions. Under 1 atm up to 700 °C they found that most pyroxenes lost H while hydration was only observed for pyroxenes rich in iron (Fe). They explained this observation by a surface redox reaction. Olivine and pyroxene are both present in the basanite employed here, which also contains 7.8% Fe (S2 Table).

We used X-ray diffraction (XRD) to detect possible changes in the mineralogy of basanite thermally treated in the absence of biomass and basanite co-pyrolyzed to form RE-biochars (9:1). The heating of pure rock powder had no effect on the speciation of silicate minerals (R450 and R750 in S2 Table). The content of all silicate minerals in the 450 °C RE-biochars was in good agreement with expected, calculated concentrations. In RE-biochars produced at 750 °C, the content of all silicate minerals fell below the expected values, while the amorphous fraction increased (Table 2); for example, the detected content of the potassium (K)-bearing mineral leucite was 50% below the expected value. We assume that X-ray diffraction was not affected by the presence of PyC as illustrated by the successful detection of calcite in pure BC750, despite a total inorganic carbon (TIC) content of < 1%. In summary, the assessment of bulk mineralogy confirmed changes induced by co-pyrolysis that were absent in thermal treatment without biomass, but did not directly prove H uptake. Further research should employ X-ray photoelectron spectroscopy (XPS) to investigate changes in binding energy in surface minerals only.

**Table 2:** Concentration of mineral species in pristine basanite rock powder (R), pure biochar (BC450, BC750) and selected rock-enhanced (RE-) biochars produced at 450 °C containing 10% rock (RE-BC450-9:1) and produced at 750 °C containing 10% rock (RE-BC750-9:1) based on X-ray diffraction (XRD) measurements. The data is presented in mass-%.

Mineral Group	Mineral Formula	R	BC450	BC450–9:1		BC750	BC750–9:1	
				expected*	measured		expected*	measured
**Quartz**	**SiO**₂	0.5	<0.1	0	0.1	<0.1	0	<LOD
**Plagioclase**	**(Na,Ca)(Si,Al)**₄**O**₈	12.9		0.7	0.8		1.1	0.8
**Clinopyroxene**	**(Ca,Na)(Mg,Fe,Al,Ti)(Si,Al)**₂**O**₆	45.6		2.6	2.5		3.8	2.6
**Olivine**	**(Mg,Fe)**₂**SiO**₄	4.5		0.3	0.2		0.4	0.3
**Nepheline**	**(Na,K)AlSiO**₄	11.3		0.6	0.6		0.9	0.8
**Sodalite**	**Na**₈**Al**₆**Si**₆**O**₂₄**Cl**₂	0.4		0	<LOD		0.0	<LOD
**Leucite**	**KAlSi**₂**O**₆	12.6		0.7	0.7		1.0	0.5
**Analcime**	**NaAlSi**₂**O**₆**•(H**₂**O)**	0.8		0	<LOD		0.1	<LOD
**Hauyne**	**(Na,Ca)**₄ ₋ ₈**Al**₆**Si**₆**(O,S)**₂₄**(SO**₄**,Cl)**₁ ₋ ₂	0.3		0	<LOD		0	<LOD
**2:1-layer silicates**	**(K,H**₃**O)(Al,Mg,Fe)**₂**(Si,Al)**₄**O**₁₀**[(OH)**₂**,H**₂**O]**	2.7		0.2	<LOD		0.2	<LOD
**Vesuvianite**	**Ca**₁₀**Mg**₂**Al**₄**(SiO**₄**)**₅**(Si**₂**O**₇**)**₂**(OH)**₄	1.3		0.1	<LOD		0.1	<LOD
**Pseudobrookite**	**(Fe³** ⁺ **,Fe²**⁺**)**₂**(Ti,Fe²**⁺**)O**₅	0.2		0	<LOD		0	<LOD
**Hematite**	**Fe**₂**O**₃	5.8		0.3	0.1		0.5	<LOD
**Ilmenite**	**Fe²** ⁺ **TiO**₃	0.3		0	<LOD		0	<LOD
**Calcite**	**CaCO**₃	0	0.2	0.2	0.1	0.9	0.8	0.1
**Apatite**	**Ca**₅**(PO**₄**)**₃**(OH,F,Cl)**	0.9		0.1	<LOD		0.1	<LOD
**Amorphous**		0	99.8	94.1	94.8	99.1	90.8	94.9

*Expected concentrations of mineral species in RE-biochars calculated based on XRD measurements of pure basanite, BC450, BC750 and the rock/ biogenic fractions of the RE-biochars. LOD = Limit of detection.

The inorganic phase is generally a more relevant contributor to elevated H:C_org_ ratios than low molecular weight hydrocarbons. The contribution of geogenic hydrogen can explain 50% of the increase in H:C_org_ observed in BC750–1:9 compared to BC750 (H:C_org_ *versus* H_Bio_:C_org_). The sorption of a dense monolayer of H_2_ to the rock surfaces (BET-surface, section 4.2) present in BC750–1:9 could explain about 5% of the remaining delta between the H_Bio_:C_org_ of BC750 and BC750–1:9. However, this can be both an over- and underestimation. On the one hand, not all mineral surfaces are exposed (pyrogenic coating) and not all sorb H_2_ well. On the other hand, small interlayer spaces of phyllosilicates (0.3-0.4 nm; most relevant for H_2_ sorption) are not accounted for in BET analysis. Hydrogen can enter pores inaccessible for N_2_. We acknowledge that there must be additional, unknown drivers contributing to elevated H_Bio_:C_org_ which were not quantified here. One possible scenario is the “*lock-in*” of water that may have formed as a product of redox reactions or in the syngas. Water may be structurally integrated in minerals (hydration) or retained in obstructed parts of the biochar pore system (section 2.3)

In summary, the elevated H:C_org_ ratio in BC750–1:9 are attributed to multiple processes that differ in their degree of evidential support. First, the contribution of geogenic hydrogen is directly quantified from bulk elemental data and accounts for 50% of the observed increase relative to BC750. Second, physical adsorption of molecular hydrogen to mineral surfaces was estimated at 5% based on the BET SSA, which carries considerable uncertainties. Third, aliphatic and less condensed (lower-aromaticity) carbon species in secondary char have much higher H:C_org_ molar ratio than the bulk biochar, though their minor mass fraction limits their quantitative contribution to the elevated H:C_org_. Fourth, the retention of water formed during redox reactions or trapped in obstructed pore domains remains a speculative and hardly quantifiable mechanism, whose contribution is, however, considered marginal. Collectively, the processes identified so far do not fully account for the elevation in H:C_org_, suggesting that additional mechanisms are at play.

### 2.2. Reduction of iron minerals during co-pyrolysis

**Fig 2:**
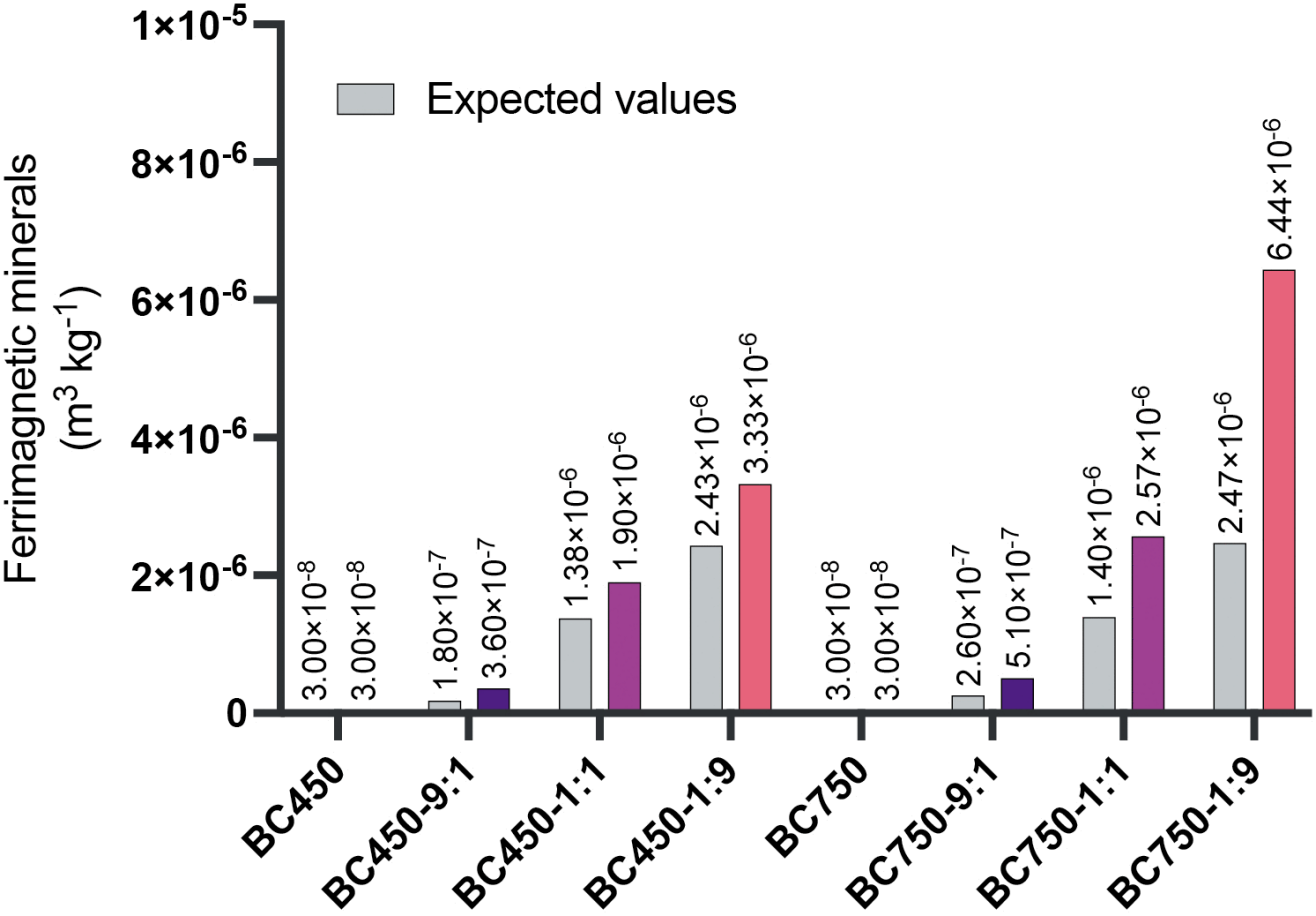
Measured content of bulk ferrimagnetic minerals in m^3^ kg^-1^ sample and expected content of bulk ferrimagnetic minerals considering their respective biogenic and geogenic fraction and ferrimagnetic properties of the latter (grey). On the X-axis: Rock-enhanced biochars produced at 450 °C (BC450) containing 10−90 wt% basanite (BC450-9:1, BC450-1:1 and BC450-1:9) and rock-enhanced biochars produced at 750 °C (BC750), containing 10−90 wt% basanite (BC750-9:1, BC750-1:1 and BC750-1:9).

The pyrolysis gas contains reducing agents such as H_2_ or carbon monoxide, which could react with mineral components and are known to reduce iron at high temperatures [53–56]. X-ray diffraction showed that the content of the ferric iron mineral hematite (Fe_2_O_3_) fell below the limit of detection as the result of co-pyrolysis, in contrast to the thermal treatment of the rock powder without biomass (S2 Table). This can be explained by the reduction of Fe(III) to Fe(II), forming, e.g., magnetite or other reduced minerals such as siderite or vivianite. The disappearance of hematite is concomitant with an increase in magnetic susceptibility (χ), which quantitatively describes the content of ferrimagnetic compounds in a sample. It is determined by measuring the extent to which a sample of known volume and mass alters a magnetic field and is expressed in m³ kg ⁻ ¹ [57]. Pure biochars contained only trace amounts of ferrimagnetic minerals (3x10^-8^ m³ kg ⁻ ¹), whereas basanite rock powder showed more than 70 times higher levels (2.25x10^-6^ m³ kg ⁻ ¹) that further increased to 2.77x10^-6^ m³ kg ⁻ ¹ upon thermal treatment at 750 °C. In RE-biochars, ferrimagnetic contents exceeded the expected mixture values, particularly at 750 °C (Fig 2). To the best of our knowledge, this is the first evidence that not only pure Fe minerals (e.g., hematite) can be reduced during the production of biochar [58], but also Fe minerals embedded in a silicate rock matrix.

### 2.3. ^1^H FFC-Relaxometry: Effects on water–surface interactions and pore domain connectivity

Nitrogen sorption isotherms and the BET method showed that RE-biochars produced at 450 °C displayed higher surface areas than expected from the arithmetic combination of biochar and rock powder surface areas (S9A,B Fig). At 750 °C, surface areas decreased progressively with increasing rock content but remained within the expected range for mixtures containing up to 50% rock, whereas the 90% rock treatment showed a marked decline (56% below the expected value). This behavior likely reflects a substantial reduction in accessible pore domains due to obstruction of pores by minerals, as suggested by Meyer zu Drewer *et al.* [24]. To complement these structural measurements with a functional characterization of water-accessible domains, we employed fast field cycling (FFC) ¹H-NMR relaxometry. This technique probes the spin–lattice relaxation of water protons within porous matrices [59]. Relaxometry provides information on water–surface interactions, thereby allowing the assessment of pore-domain accessibility and connectivity in porous materials such as biochars.

**Fig 3:**
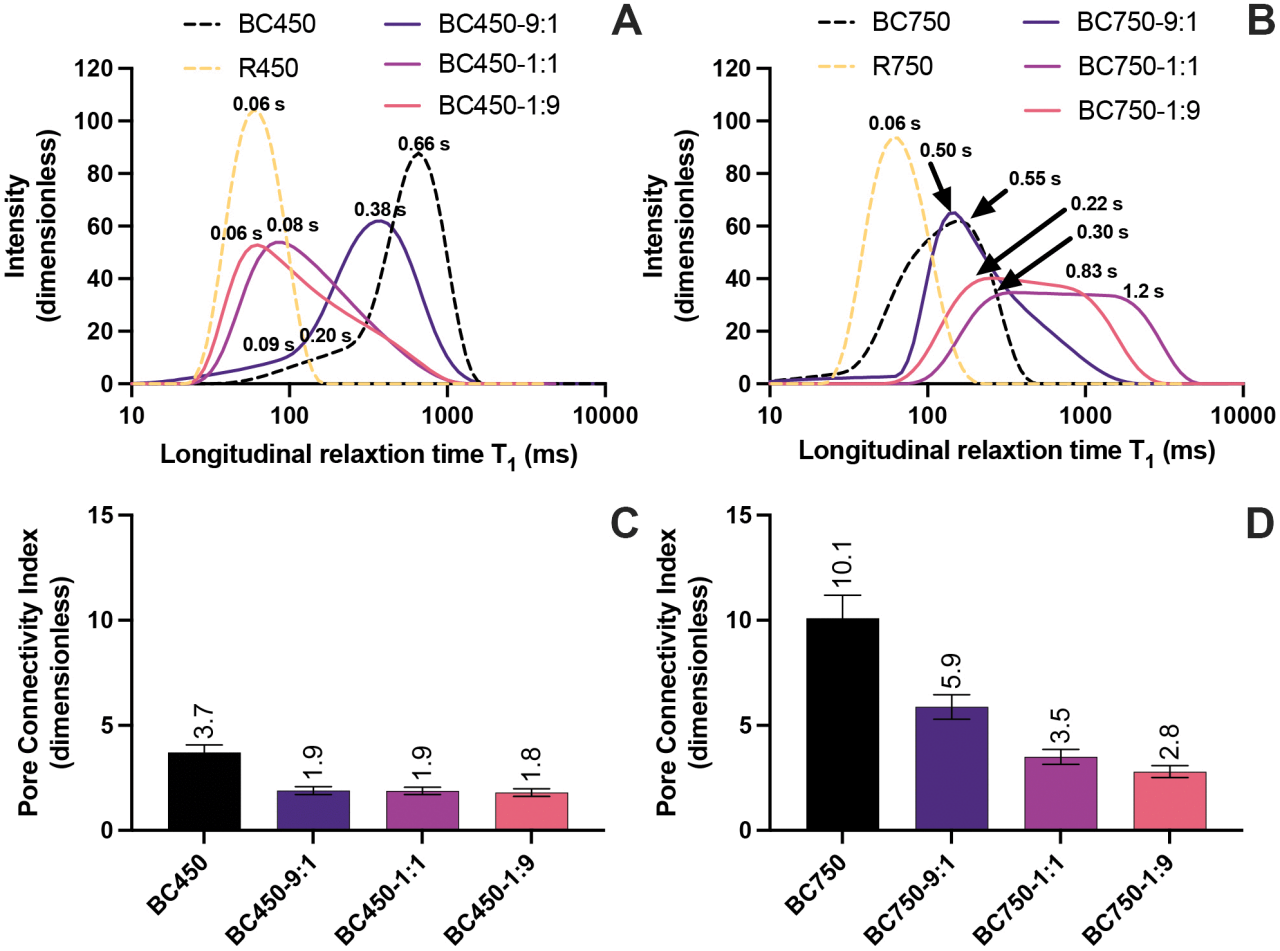
(A + B): Distribution histogram of measured longitudinal relaxation times (T_1_) of hydrogen during fast field cycling nuclear magnetic resonance (FFC-NMR) relaxometry. Logarithmic scaled X-axis in milliseconds and in-figure labels in seconds. (C + D): Dimensionless pore connectivity index (PCI) based on T_1_ relaxation times, presented as mean ± standard deviation. Left: (Rock-enhanced) biochars pyrolyzed at 450 °C (BC450) containing 10-90% basanite (BC450-9:1, BC450-1:1 and BC450_1:9). Right: (Rock-enhanced) biochars pyrolyzed at 750 °C (BC750), containing 10-90% basanite (BC750-9:1, BC750-1:1 and BC750-1:9). Measurements on pure basanite rock powder, after thermal treatment at 450 °C (R450) and 750 °C (R750) respectively are indicated as yellow dashed lines in panel A and B.

The relaxation patterns in Fig 3 reveal three effects of co-pyrolysis with rock powder. First, the shift of T_1_ distributions reflects HTT-dependent changes in the balance between water–surface and water–water relaxation. At 450 °C, rock addition shifts T_1_ toward shorter values, indicating that water is more strongly bound to biochar and mineral surfaces. In contrast, at 750 °C, rock addition shifts T_1_ toward longer values. This in turn is indicative for increasingly bulk-like relaxation environments where water interacts less strongly with biochar and mineral surfaces (i.e., more bi-polar water-water connections in larger pores). The observed opposite trends align with the HTT-dependent restructuring of accessible pore domains inferred from BET measurements, where the SSA increased with rock content at 450 °C but decreased at 750 °C. Second, the marked decrease in the pore connectivity index (PCI) indicates reduced exchange between relaxation domains, implying that water mobility becomes increasingly confined to domains that exchange less efficiently with each other. This observation implies a generally reduced mobility of liquids and potentially gases in RE-biochar. Third, in RE-biochars produced at 750 °C, the alteration of T_1_ distributions is consistent with the formation of mixed-valence Fe(II)/Fe(III) oxides such as magnetite, generated through the reductive transformation of Fe-oxides during pyrolysis. Such paramagnetic phases can contribute to the overall relaxation behavior, and may locally increase relaxation efficiency (i.e., shorter T₁) Accordingly, the net shift toward longer T₁ at 750 °C is interpreted primarily as a pore-domain/ accessibility effect (more bulk-like water environments), with magnetic phases acting as an additional, superimposed contribution [57,60,61].

Altogether, these effects highlight a chemically active interfacial environment during the co-pyrolysis, leading to the restructuring of pore domains compared to pure biochar and mineralogical transformations that shape porosity and surface properties. Given these effects, it is plausible that, at sites enriched in reactive or partially reduced metals, a fraction of interfacial carbon may experience interactions stronger than those expected from simple physisorption, potentially including the formation of metal–carbon (M–C) linkages and complexes. Possible examples include carbides such as Fe_3_C [62]. While such interactions could in principle contribute to the modified relaxation behavior, they cannot be detected by FFC-NMR alone and would require complementary high-resolution spectroscopic or microscopic analyses for verification. At this stage, M–C linkages can therefore be regarded as a possible, yet unconfirmed, additional contribution to the overall interfacial relaxation environment.

### 2.4. Carbon speciation at the biochar-mineral interface

**Fig 4:**
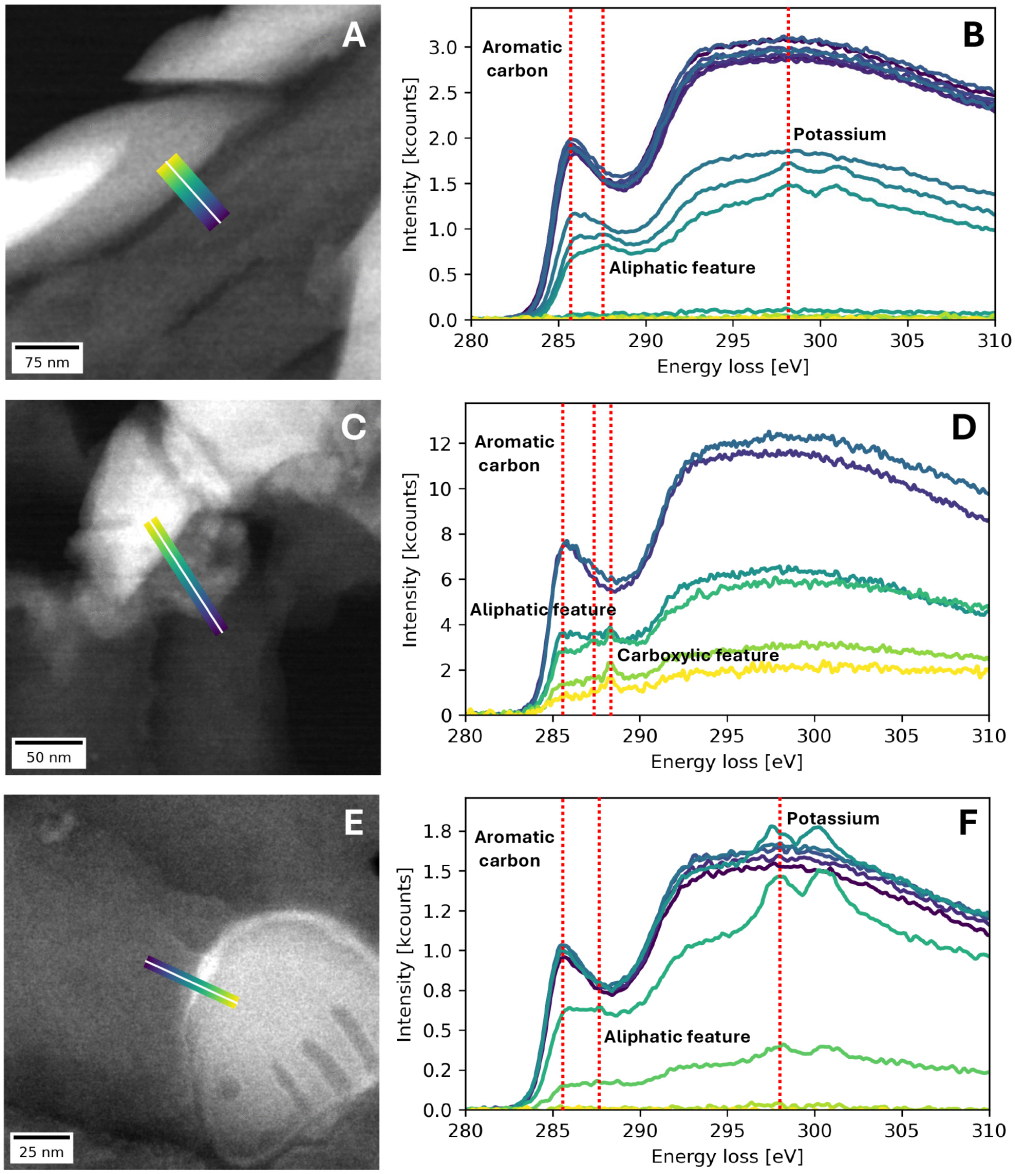
Left panels (A,C,E): Scanning transmission electron micrographs obtained in high-angle annular dark field mode (HAADF), showing ultrathin cross section of a BC750-1:1 pellet. Mineral particles (white) are embedded in the surrounding biochar matrix (dark) with an indicated measurement transect (color-coded) spanning the pure biochar (blue to purple) over the mineral-biochar interface (green to turquoise) into the rock particle (yellow to light green). Right panels (B,D,F): Corresponding electron energy loss spectroscopy (EELS) spectrograms obtained along the measurement transect. Note that the Si-O peaks are cropped from the spectra. Three additional spectrograms are shown in S10 Fig.

Scanning transmission electron microscopy coupled to electron energy loss spectroscopy (STEM-EELS) was used to resolve elemental composition and carbon speciation in BC750–1:1 at the nanometer scale (Fig 4, S10 Fig). Six EELS transects crossing from biogenic components of RE-biochar into adjacent rock consistently showed the K edge of aromatic C at 284.9–285.5 eV [63] transitioning to the characteristic Si-O peak at 540 eV [64] within the mineral phase (Si-O peak cropped from Fig 4). These measurements revealed a distinct carbon speciation at the biochar-mineral interface. Aliphatic carbon features (σ(C–H) and σ(C–C) at 287–288 eV [65,66] were detected in 4 of 6 scans, while carboxylic features at 288.6 eV [63,67] occurred in 2 of 6 scans. Such aliphatic and oxygenated signals align with the known composition of volatile pyrolysis intermediates like furans, phenols, alkanes, alkenes or alkynes, and support the idea of a pyrogenic coating of mineral surfaces. Basanite is a non-acidic aluminosilicate rock, composed mainly of plagioclase, pyroxene, and nepheline. These minerals provide negligible Brønsted acidity and therefore behave similarly to other non-acidic silicate surfaces, which are known to sorb and stabilize radicals and oxygenated vapors rather than drive catalytic transformations [68,69]. Accordingly, the interfacial carbon detected here is most likely derived from adsorption and consistent with the thin pyrogenic coating [24]. The presence of organo-mineral bonds as suggested above could not be confirmed, but the limited number of STEM-EELS scans can neither refute their existence. The sorption of such low-molecular compounds with a high H content (e.g., H:C ≈ 2 in alkanes) – or more complex pyrogenic hydrocarbons is a minor contributor to elevated H:C_org_ ratios.

**Fig 5:**
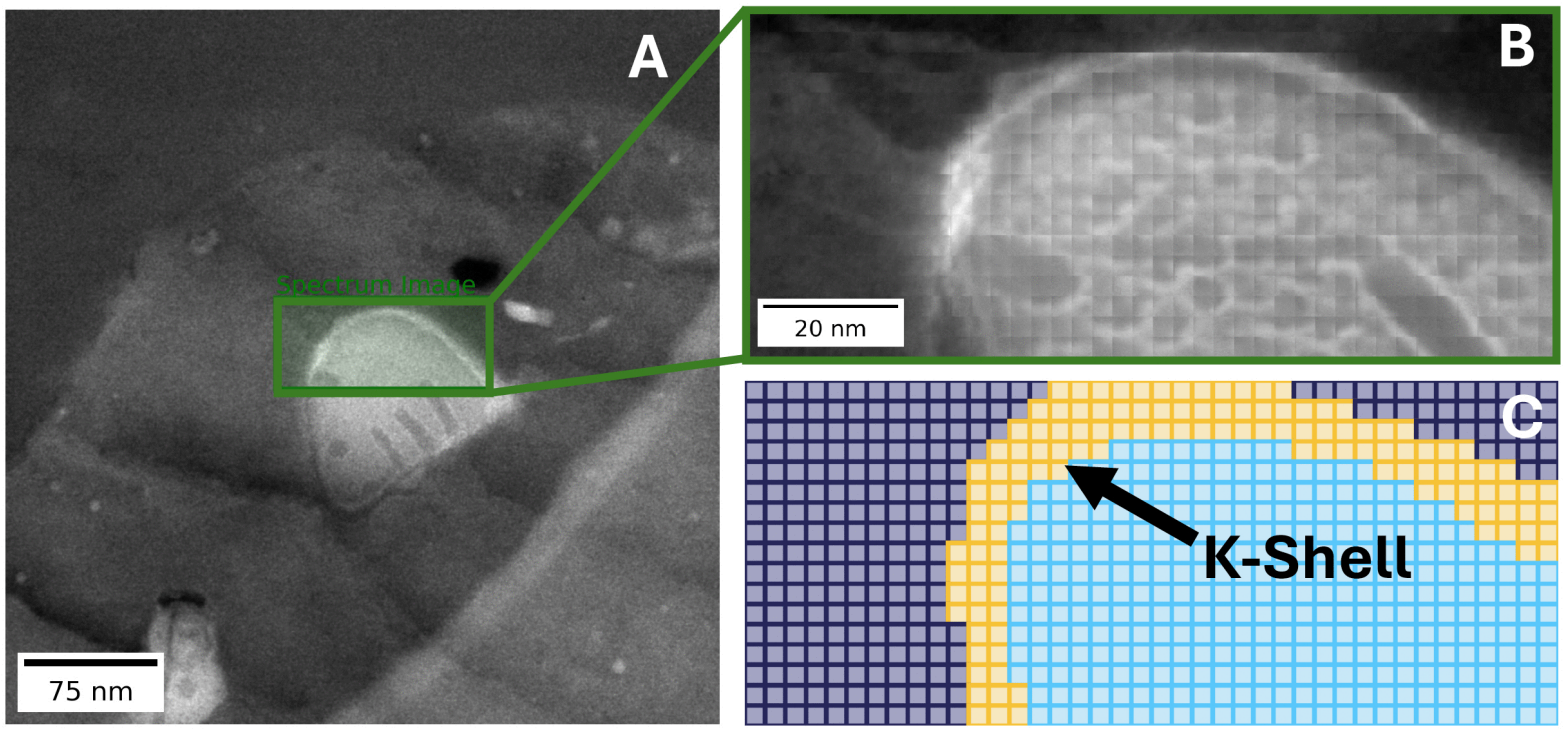
(A): Micrograph showing an ultrathin cross section of a BC750-1:1 pellet, with clipping (B) subjected to Electron Energy Loss Spectroscopy mapping of elemental distributions. (C): *Mapping of the distribution of the dominating elements: silicon-dominated rock particle (light-blue), carbon-dominated biochar matrix (dark-blue), potassium-rich shell (yellow).*

Elevated concentrations of K were identified at the biochar-mineral interface in 3/6 scans (297–300 eV) [70] and a 2D EELS mapping revealed a *potassium shell* at the mineral surface (Fig 5). Beam-induced K migration [71] was excluded experimentally (Inversed beam trajectory; S10B Fig). Enrichment of biogenic ash around rock particles could in principle produce such a shell, but this would also result in proportional enrichment of other cations, which was not observed. Instead, pyrogenic free radicals [72,73] may mobilize K from the outer layers of K-rich basanite minerals such as nepheline or leucite, whose heterogeneous distribution at the micrometer scale – as confirmed by scanning electron microscopy coupled with energy dispersive X-ray spectroscopy (SEM-EDX, S11 Fig) – likely explains the occurrence of K enrichment in only 3 of 6 STEM transects. This process may lead to enhanced alkalinity production due to a stronger release of basic cations (such as K^+^) in short-term weathering experiments using RE-biochar.

### 2.5. Matrix-effects of inorganic phases on biochar analysis

The inorganic constituents of RE-biochar differ fundamentally from aromatic carbon moieties. Where mineral enrichment is high (e.g., 90% rock), inorganic constituents contribute a relevant volume-fraction to the RE-biochar. In such a case, interface regions are widely distributed and may become the spatially dominating surface in the system. Accordingly, it is necessary to critically examine whether established methods of biochar analysis can be applied to RE-biochar. It has been shown that the main and trace elements in RE biochar can be reliably quantified after chemical digestion, even though X-ray fluorescence is commonly used for rock [24]. The present study, however, revealed distortions in thermal and optical analytical techniques.

Following TGA (50−1000 °C), RE-biochars produced at 450 °C showed higher BC_1000C_ fractions than BC450, while BC_1000C_ for BC750–1:9 was zero. These findings cannot be reconciled with the results of HyPy and BCPA, which in turn provided a consistent picture. A likely source of error is the challenge to obtain a representative sample at the milligram-scale from a material with large density contrasts (biochar: ~ 0.2 kg L^-1^; basanite: ~ 2.6 kg L^-1^). If the actual ash content in the 5 mg subsample for TGA deviates strongly from the nominal value used for daf correction, mass loss and BC_1000C_ fraction may be over- or underestimated [24].

Matrix effects from the higher thermal conductivity and heat capacity of basanite compared to biomass and biochar may further distort apparent thermal stability [24]. Equivalent PPMs could not be reliably analyzed due to subsampling uncertainty at the milligram scale [24], and there was not enough biochar available for a mixture in the gram range followed by grinding in the vibratory ball mill. Therefore, the results of the thermal analysis of the RE-biochars can be unreliable and must be interpreted with caution. Demineralization of high-ash biochar samples, e.g., hydrofluoric acid treatment, is one option to be considered for the removal of silicates. It was shown that pre-analysis demineralization of samples can increase the predictive power of, e.g., elemental analysis and H:C_org_ molar ratio to forecast actual aromaticity [74].

**Fig 6:**
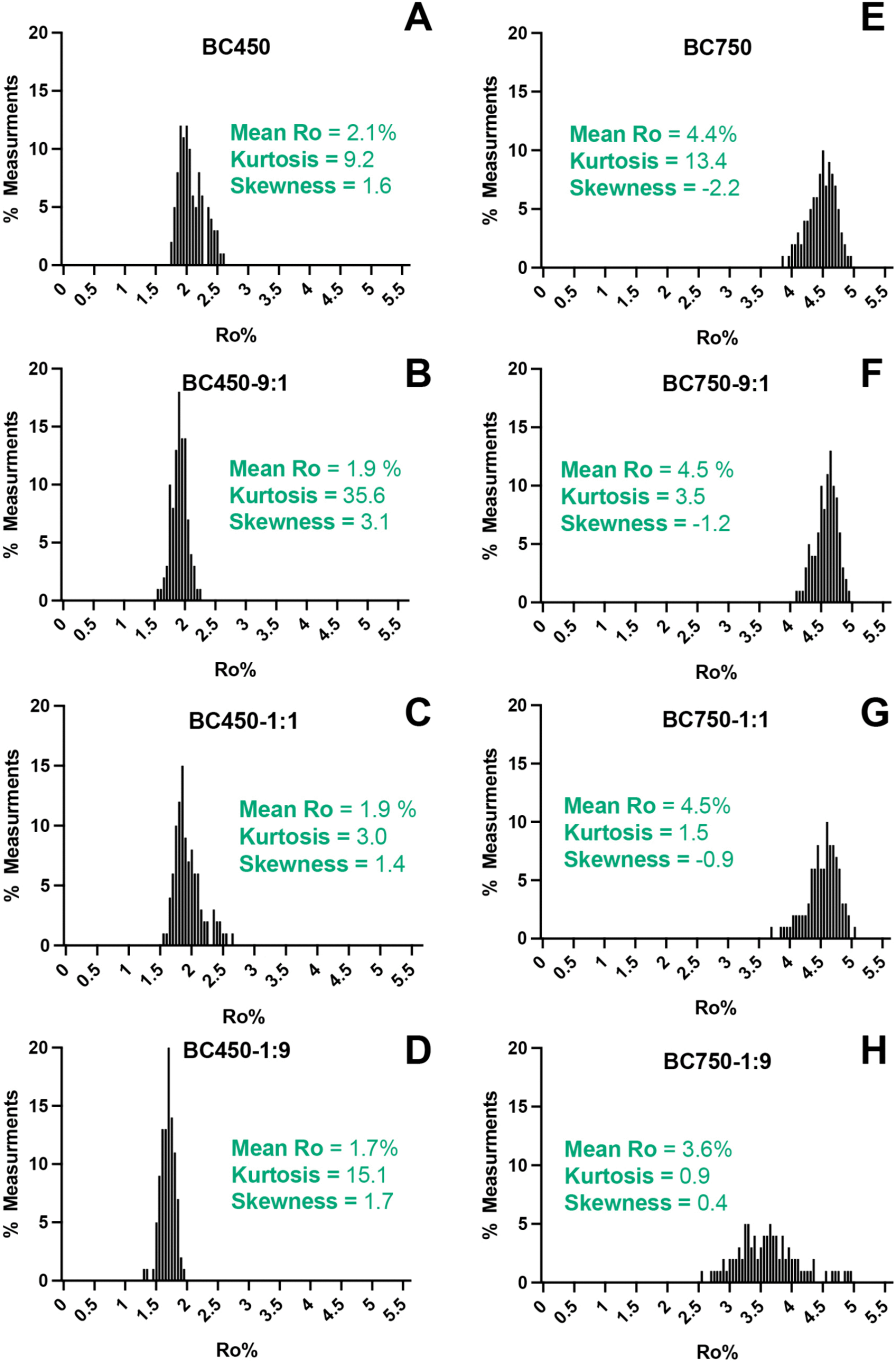
Histograms of random reflectance (Ro%) distributions obtained from n = 500 measurements per sample. Left column: Rock-enhanced (RE-)biochars produced at 450 °C, containing (A): 0% (BC450), (B):10% (BC450-9:1), (C): 50% (BC450-1:1) or (D): 90% basanite rock powder (BC450-1:9). Right column: RE-biochars produced at 750 °C, containing (E): 0% (BC750), (F):10% (BC750-9:1), (G): 50% (BC750-1:1) or (H): 90% basanite rock powder (BC750-1:9). Indications in green: Mean Ro%, Kurtosis (Gaussian distribution = 3, leptokurtic >3 and platykurtic <3) and Skewness (Gaussian distribution = 0, right skewed >0 and left skewed <0).

Random reflectance is increasingly used in biochar characterization [35,75–77]. For BC450, a mean Ro of 2.1% was recorded, which decreased to 1.7-1.9% in RE-biochars (Fig 6) and Ro% distributions were increasingly leptokurtic at 50−90% rock (narrowing of the curve = clustering of data around mean) with kurtosis of 1.7-3.0 (gaussian distribution = 3). This likely reflects a more homogeneous heating due to improved thermal conductivity of the rock–biomass mixture. For BC750 mean Ro was 4.3%, which increased to 4.5% in BC750–9:1 and BC750–1:1 and decreased to 3.6% in BC750–1:9 with increasingly platykurtic distribution (spreading of the distribution curve = larger standard deviation) with increasing rock content. Generally, HTT 450 °C samples were right skewed, i.e., the population had a lower median than mean, while HTT 750 °C samples were rather left skewed. These strong effects of rock-enhancement on Ro% distributions, despite nearly unchanged aromaticity (c.f. BC_HyPy_, BPCA-6, FTIR, Raman) highlights that a high ash content can considerably impact Ro determination. It remains speculative whether this is due to the influence of the rock on the reflective properties or whether a proportion of the aromatic carbon now interacts with the rock in such a way that these structures can no longer be identified as macerals under a light microscope and thus not contribute to Ro. Therefore, Ro-derived metrics (e.g., persistence indices or carbonization temperatures) and mean Ro values of skewed distributions must be interpreted with caution. Both TGA and Ro highlight limitations of biochar analysis when applied to RE-biochars or high ash biochars in general. Future research but also routine analysis, such as for the European Biochar Certificate (EBC) [78], should explicitly account for these matrix effects in experimental design, sample preparation and data interpretation.

## 3. Conclusions

Co-pyrolysis of biomass with silicate rock powder was proposed as an avenue to synergistically combine PyCCS- and ERW-based carbon dioxide removal in one composite material. Geogenic metals (incl. AAEM) can catalyze biomass pyrolysis and potentially engage in the formation of organo-metallic bonds (e.g., Fe-C). However, the impact of AAEM in our study is limited, as the y_c_ and PyC speciation (aromaticity as indicated by HyPy and BPCA-marker assessment) of RE-biochars was not altered compared to pure biochar. This suggests that up to a HTT of at least 750 °C, AAEM embedded in crystalline structures of basanite rock remain largely inactive as pyrolysis catalysts. The identification of organo-metallic bonds would require dedicated spectroscopic evidence, which was beyond the scope of this study and thus was neither confirmed nor refuted. Rock-enhancement does not impact the environmental persistence of RE-biochars – as indicated by BC_HyPy_ content and BPCA markers.

It was demonstrated that H:C_org_ molar ratio of RE-biochar must be interpreted with caution. The inorganic phase can both contribute geogenic H and is able to sorb biogenic H. This is distorting the bulk H:C_org_ ratio of the RE-biochar, which can no longer be interpreted independent of the inorganic phase. Also, other analytical approaches like random reflectance and thermal analysis may be compromised by matrix-effects where rock powder was added at high rates. Matrix-effects require urgent consideration during experimental design and data interpretation, not just for RE-biochar, but also for other high ash biochars. When no comparable low-ash biochar is available as a reference, as it is the case, e.g., for sewage sludge, hydrofluoric acid treatment should be tested to remove mineral components prior to elemental analysis.

Mineral surfaces exposed to syngas experience several alterations during co-pyrolysis. These include the reduction of iron-oxides, the locally discrete accumulation of K, and the pyrogenic coating with conductive secondary char. These observations provide first evidence that silicate rock particles can be modified by co-pyrolysis with biomass. These alterations may be exploited in a context of ERW (faster weathering, cf. [26]), agriculture (increased availability of nutrients, e.g., K-phosphates) and functionalized biochars (improved sorbents) – however all three fields require further research, which also needs to expand on how rock additions impact the agronomic performance of biochar.

## 4. Materials and methods

### 4.1. Biomass preparation and pyrolysis

Spruce wood shavings (*Picea abies* L.) were sourced from Allspan German Horse GmbH, Karlsruhe, Germany (50.7% C, 0.5% ash; S1 Table). Basanite rock powder (“Eifelgold”; particle size range 0–250 µm) was obtained from Provinzial-Basalt- u. Lavawerke, Sinzig, Germany and characterized by X-ray diffraction, X-ray fluorescence, laser granulometry and extraction in ultra-pure water as described elsewhere [24]. The basanite’s SSA was measured as described in section 4.2. Both, pure biomass and homogenized mixtures of biomass with fine rock powder were pelleted at 6 mm without additional binders using a WK230 pellet press (Evertec, Dieburg, Germany). Afterwards, the pellets were dried (12 h at 60 °C), and fine material (< 6 mm) was removed by sieving.

The required feedstock mixing ratios to obtain RE-biochars with nominal 10%, 50% or 90% rock content, were calculated based on mass-yield (y_m_) measured during production of pure biochar at 450 °C (BC450; y_m_ = 25.9%) and at 750 °C (BC750; y_m_ = 18.9%). The mass of the basanite was constant during pyrolysis, which was also verified by thermo-gravimetric analysis (TGA) of pure basanite. As some rock powder might got lost during pelleting and sieving, the actual biogenic (f_bio_) and rock mass fraction (f_rock_) of the feedstock was re-calculated following equation 1 and 2 (Table 3).

**Table 3:** Composition of feedstock to produce rock-enhanced biochar (BC) at 450 °C (450) or 750 °C (750), containing 10% (9:1), 50% (1:1) or 90% (1:9) basanite rock powder after pyrolysis.

#	Sample ID	Biogenic fraction (%)	Rockfraction (%)	C_org_(%)	Biogenic ash(%)	Total ash(%)
1	BC450	100	0	50.6	0.4	0.4
2	BC450–9:1	97.9	2.1	49.5	0.4	2.5
3	BC450–1:1	75.9	24.1	39.2	0.3	24.4
4	BC450–1:9	28.8	71.2	15.0	0.1	71.3
5	BC750	100	0	50.6	0.4	0.4
6	BC750–9:1	98.6	1.40	50.4	0.4	1.8
7	BC750–1:1	84.0	16.0	42.0	0.3	16.3
8	BC750–1:9	39.7	60.3	18.1	0.1	60.4


$$\begin{array}{c} f_{rock}(\%)=Ash\;(\%)-C_{org}(\%)*r \end{array}$$ (1)

$$\begin{array}{c} f_{bio}(\%)=\text{1}\text{0}\text{0}-f_{rock}(\%) \end{array}$$ (2)

With ash (%) being the ash content and C_org_ (%) the organic carbon content of the feedstock mix. The factor r describes the ratio of C_org_ to ash in the pure biomass (s1). The same procedure was applied to calculate f_bio_ and f_rock_ for RE-biochars, assuming no mass loss of rock powder during pyrolysis and no impact of rock powder on biochar dry and ash free (daf) yield.

Feedstock transport-rates (t_f_ in g min^-1^) through the non-heated pyrolysis reactor were determined for each treatment by measuring material output in 10 min intervals (*n* = 6). In the same manner, biochar transport-rates (t_b_ in g min^-1^) through the heated pyrolysis reactor were determined in 30 min intervals (*n* = 3). Pyrolysis was performed at 450 °C and 750 °C, respectively, with a residence time of 15 min using a PYREKA research pyrolysis unit equipped with a continuously operating auger reactor (PYREG GmbH, Dörth, Germany) under Nitrogen flow of 2 L min^-1^ [24,27]. The PYREKA operated in one continuous process per treatment.

The ratio of feedstock to biochar output during the first hour (equivalent to four reactor residence times) and after the feeder ran empty, was excluded from transport rate calculations and therefore from calculation of y_m_ and y_c_. The biochar mass yield was calculated as follows:


$$\begin{array}{c} y_{m}(\%)=\frac{t_{b}}{t_{f}}*\text{1}\text{0}\text{0} \end{array}$$ (3)

With y_m_ being the pyrolysis mass yield as % of dry weight feedstock converted to dry weight biochar, t_f_ the mean transport-rate of the feedstock (g min^-1^) and t_b_ the mean (RE-)biochar transport-rate (g min^-1^). Exceptions were BC450–1:9 and BC750–1:9, here, due to the limited material volume, no transport-rate was determined but the total mass before and after pyrolysis measured. Further, the coefficient of variation (%CV) in t_b_ was calculated as a proxy for the variability of the continuous production process and resulting y_c_.

The y_c_ was calculated as follows:


$$\begin{array}{c} y_{c}(\%)=\frac{y_{m}*C_{org(BC)}}{C_{org(f)}} \end{array}$$ (4)

With y_m_ being the mass yield of pyrolysis process (%), C_org(f)_ the C_org_ content of the feedstock and C_org(BC)_ the C_org_ content of the produced (RE-)biochar.

The (RE-)biochar production during the ramp-up/down time as described above was discarded not to be included in any analytical sample. The remaining production was pooled and thoroughly mixed. While many of the pellets were broken into smaller fragments by the moving parts in the reactor, ten intact pellets were randomly selected and set aside for microscopy. The remaining (RE-)biochar was milled in an impact mill with 2 mm sieve. Due to the brittle nature of the material, a fine powder was produced this way. The particle size distribution of comparable biochars after milling was quantified by laser granulometry (Sympatec Helos KFMagic, Sympatec GmbH, Clausthal-Zellerfeld, Germany) and is typically one order of magnitude smaller than the sieve size [27] (> 90 mass-% at < 200 µm for a 2 mm sieve).

To supply larger quantities of (RE-)biochar to other follow-up experiments (not subject to this publication), a second batch of all treatments was produced. To this end, the same raw materials were used (S3 Table), pelleted by Nature Power Pellets (Wolferstadt, Germany) and pyrolyzed on the PYREKA under same process conditions as described above. The pellets produced by Nature Power Pellets had the same dimensions but a higher density. Biochar and RE-biochars from the first production batch were subject to all analytics described in section 4.2 while materials from the second batch were only subject to basic characterization (S4 Table) with additional calculation of y_m_, y_c_, f_bio_ and f_rock_.

### 4.2 Biochar analysis

All (RE-)biochars were analyzed following the analytical guidelines of the European Biochar Certificate [78] by Eurofins Umwelt-Ost GmbH (Bobritzsch-Hilbersdorf, Germany). The C_org_ content of the daf biochar (C_org_daf_) was calculated as follows


$$\begin{array}{c} C_{org_{daf}}(\%)=\frac{C_{org}(\%)}{\text{1}\text{0}\text{0}\%-\text{Ash}(\%)}*\text{1}\text{0}\text{0}\% \end{array}$$ (5)

With C_org_(%) being the organic carbon content and ash (%) the total ash content of the (RE-)biochar.

The hydrogen to organic carbon molar ratio (H:C_org_) of the bulk sample was corrected for geogenic hydrogen using equation 6.


$$\begin{array}{c} {H_{Bio}:C}_{org}=\frac{{H\%}_{Bulk\;}-(f_{rock}*{H\%}_{rock})\;}{M_{H}}*\frac{M_{C}}{Corg\%\;} \end{array}$$ (6)

With H:C_orgBio_ being the hydrogen of the biogenic fraction to C_org_ molar ratio, i.e., the bulk ratio corrected for geogenic hydrogen, %H_Bulk_ and C_org_ content of the bulk sample, f_rock_ the rock fraction of the sample, H%_rock_ the hydrogen content of the basanite and M_C_ and M_H_ the molar mass of carbon and hydrogen. The carbon content of the rock was below the detection limit, and we assume that all rocks relevant to ERW do not contain organic carbon.

Solid state electrical conductivity (SEC) was measured using a Black Gauss I conductivity meter [79] while applying 5 t pressure (equivalent to 160 MPa) to the sample. The mineralogical composition of selected RE-biochars was measured by XRD on a D8 Advance (Bruker, Billerica USA) performed by QMineral (Heverlee, Belgium).

Thermogravimetric analysis (TGA) was conducted using an STD 650 TG-DSC system (Waters, New Castle, United States of America). Dry samples (5 mg) were heated at open, pre-weighed alumina crucibles. The temperature was then increased from 50 °C to 1000 °C at a rate of 10 °C min ⁻ ¹ under N_2_ atmosphere with a flow of 50 mL min ⁻ ¹. The recorded total weight loss was adjusted for the sample’s total ash content (including rock content) to represent the weight loss of daf biochar. The fraction of biochar that remained resistant to thermal degradation up to 1000 °C is referred to as BC_1000C_.

Benzenepolycarboxylic acids marker (BPCA) were extracted and analyzed in duplicate following Glaser *et al.* [80] and Brodowski *et al.* [81]. In brief, the (RE)-biochar samples were digested using trifluoroacetic acid to remove metal ions and organic matter including carbonates and C_org_ other than those in aromatic moieties. Then the samples were oxidized in a pressurized vessel using nitric-acid. The digests were purified from remaining polyvalent cations using exchange resins. After freeze-drying, BPCAs were trimethylsilyl derivatized and analyzed by gas chromatography connected with a flame ionization detector (GC-FID) (GC-2010 Shimadzu, Kyoto, Japan).

Two types of analytical pyrolysis were performed. Firstly, hydropyrolysis (HyPy) was performed according to Meredith *et al.* [32]. In brief, 300 mg were mixed with 10 wt% ammonium molybdate-tetrahydrate as a catalyst. The samples were then heated in a hydrogen purged reactor under 150 bar pressure, first from ambient temperature to 250 °C at a rate of 300 °C min ⁻ ¹, and subsequently from 250 °C to 550 °C at 8°C min ⁻ ¹, where samples were held for 2 minutes. The residual sample was weighed and analyzed for its C_org_ content, referred to as BC_HyPy_ and presented in percent of the sample’s initial C_org_. Secondly, using a HAWK sequential-pyrolyzer with coupled TOC analyzer (Wildcat Technologies, Humble, Texas USA). Here, 10 mg were inserted into the pyrolyzer preheated to 300 °C and held for 3 min under helium atmosphere. The mass of the carbon released as CO and CO_2_ was continuously measured and is referred to as S1 and relates to free hydrocarbons such as oils [76]. Then the sample was heated at 25 °C min^-1^ to 650 °C with the mass of volatilized carbon summarized as S2. After cooling to 150 °C, the atmosphere was changed to oxygen, and the sample was re-heated to 850 °C at 25 °C min^-1^ oxidizing the residual carbon [82]. S1 + S2 was defined as the reactive, labile carbon and the residual carbon was considered unreactive under the conditions applied [35,76].

Fourier transformed infrared spectroscopy was conducted using attenuated total reflectance (ATR) mode with an Invenio X FT-IR Spectrometer (Bruker Corporation, Billerica, USA). Infrared absorbance was determined from 60 consecutive scans, covering a wavenumber range of 400–4000 cm ⁻ ¹. The absorbance spectrum was then smoothed and baseline-corrected using the software Spectragryph v.1.2.26.1.

Raman spectroscopy was conducted under a LabRAM HR 800 Raman microscope (HORIBA Europe GmbH, Oberursel, Germany), which was coupled to an BX41 light microscope (Olympus Corporation, Tokyo, Japan). All measurements used a 532 nm laser (5 mW), which scanned a 100x100 µm area. Spectral acquisition time was 10 s. Five measurements were conducted per treatment and spectra converted D/G intensity- and area ratios. The baseline correction was done using the LabSpec 6 software (Horiba) and consecutive band-fitting according to Ferrari & Robertson (2000) [83] using a Lorentzian line shape (D-band) and a Breit-Wigner-Fano line shape (G-band) with a self-made Matlab-script.

Fast field cycling ^1^H nuclear magnetic resonance relaxometry (^1^H FFC-NMR, i.e., ^1^H Relaxometry) was performed following Conte (2021) [59]. Briefly, samples of 1 g sample was weighed in a 10 mm NMR tubes, then water was added in 2.5:1 ratio. The mixture was vortexed for 5 minutes and left to settle for 24 h. Measurements were conducted on a Stelar Smartracer Fast-Field-Cycling Relaxometer (Stelar SRL, Mede, Italy) set to 25 °C. The proton spins were polarized at a polarization field (B_POL_) corresponding to a proton Larmor frequency (n_L_) of 10 MHz for a period (T_POL_) of about five times the T_1_ estimated at this frequency. After each B_POL_, the magnetic field intensity (B_RLX_) was set at the proton Larmor frequency, n_L_, of 1 MHz. The period τ, during which B_RLX_ was applied, has been varied on 32 logarithmic spaced time sets. Free induction decays (FID) at each τ value were recorded following a single ^1^H 90 ° pulse applied at an acquisition field (B_ACQ_) corresponding to n_L_ of 7.2 MHz. A time domain of 100 μs sampled with 512 points was applied. Field-switching time was 3 ms, while spectrometer dead time was 15 μs. For all the experiments, a recycle delay of 2 s was used. All decay curves acquired were analyzed by the UPEN algorithm to obtain the relaxograms [84,85]. Relaxograms were finally used to calculate the Pore Connectivity Index (PCI) [86] (additional S12 Text).

For SEM, RE-biochar pellets were mounted on an aluminum sample holder using carbon pads (Plano GmbH, Wetzlar, Germany) and sputter-coating them with gold (3–5 nm) using a Cressington 108auto (TESCAN GmbH, Dortmund, Germany). Analysis was conducted with a JSM-6610 LV (JEOL Ltd., Tokyo, Japan) at a working distance of 11 mm and acceleration voltage of 15 kV. Backscattered electron images (BES) were acquired, and Energy-dispersive X-ray (EDX) analysis of the same clippings was carried out using a 20 mm² Oxford X-max detector (Oxford Instruments, Abingdon, United Kingdom).

For Scanning Transmission Electron Microscopy (STEM) with Electron Energy Loss Spectroscopy (EELS) specimens, Re-biochar samples (< 3 mm), were embedded in epoxy resin (Spezifix 40, Struers, Willich, Germany). Ultrathin sections of 50 nm were prepared using a Leica EM UC6-NT ultramicrotome (Leica Microsystems, Vienna, Austria) equipped with a 35° ultra diamond knife (Diatome, Biel, Switzerland). The sections were transferred onto lacey carbon-coated copper grids using a Perfect Loop (Diatome). The measurements were performed using a monochromated probe corrected FEI Titan G2 60–300 STEM-microscope with a Schottky-type X-FEG electron source operated at 300 kV. The microscope was equipped with a Gatan Imaging Filter, Quantum including a direct electron detection camera (K2 Summit) and a CCD camera (Ultrascan1000) with dual-EELS capability. The microscope was operated in monochromated mode with a semi-convergence angle of 15.6 mrad and a beam current of about 200 pA, resulting in a spatial resolution of 0.12 nm and an energy resolution at full-width at half maximum of 0.3 eV. The measurements were performed at a temperature of −160 °C using a cryo TEM-holder (Mel-Build, Fukuoka, Japan). The high angular annual dark field (HAADF) detector was used for micrographs acquisition.

Gas adsorption was performed using nitrogen isotherms at 77 K on an AUTOSORB-IQ volumetric sorption device (3P-Instruments GmbH & Co KG, Odelzhausen, Germany). Samples of 0.1-0.2 g biochar were degassed at 150 °C for 12 h with a final vacuum of <10 ⁻ ² Pa applied before analysis. Surface area (Brunauer-Emmett-Teller method) was calculated following the guidelines of DIN ISO 9277:2014. The pore volume distribution is inferred from the total pore volume determined at P/P_0_ = 0.99 (Gurvich’s law) and measured adsorption isotherms according to the density functional theory (DFT) [87].

The magnetic susceptibility was measured on 6.4x10^-6^ m^3^ cubes of manually compressed material. Measurements were conducted in a 200 A m^-1^ field at low (976 Hz) and high (15616 Hz) frequency (χlf and χhf) using a MFK1-FA Kappabridge (AGICO Inc., Brno, Czech Republic). The frequency dependent magnetic susceptibility was calculated as [(χlf—χhf)/χlf] × 100 [57,60].

Random Reflectance (Ro%) was measured on (RE)-biochar fragments between 63 and 1000 µm, that were embedded in an epoxy-resin and polished (ISO 7404−2 2009). White light reflectance measurements (ISO 7404−5, 2009) were carried out using a Zeiss Axio Imager II microscope (Zeiss, Oberkochen, Germany) with integrated Discus-Fossil system (Hilgers Technisches Buero, Königswinter, Germany). Measurements were conducted manually on *n* = 500 randomly selected, exposed macerals, with each measurement covering an area of 0.3 µm². Reflectance measurements were calibrated using KB N Lasf1.317 and KB cubic zirconia 3.11 standards.

### 4.3. Statistical considerations

Rock-enhanced biochars were produced in a single continuous batch, with RE-biochar yield recorded at regular intervals and the coefficient of variation of f_b_ and y_c_ calculated. In general, analyses were conducted on representative samples without replication (*n* = 1). Depending on the analytical method varying numbers of repeated measures were conducted per sample (e.g., Raman spectroscopy with *n* = 5, FTIR with *n* = 60, and random reflectance with *n* = 500). Mean, median, skewness and kurtosis of each Ro distribution were calculated using Microsoft Excel.

## Supporting information

S1 Table Biomass analysis. (PDF)

S2 Table Rock characterization. (PDF)

S3 Table Feedstock composition (2^nd^ batch). (PDF)

S4 Table Biochar basic characterization (2^nd^ batch). (PDF)

S5 Fig ATR-FTIR absorbance spectra. (PDF)

S6 Fig Raman spectra (D/G ratios). (PDF)

S7 Fig Graphical Abstract. (TIF)

S7 Table Biochar main and trace elements (1st batch). (PDF)

S8 Table Hydrogen in small hydrocarbons. (PDF)

S9 Fig Specific surface area and pore volume distribution. (PDF)

S10 Fig STEM-EELS transects with inverted beam trajectory. (PDF)

S11 Fig SEM/EDX potassium distribution mapping. (PDF)

S12 Text Methods – Pore Connectivity Index. (PDF)
