# Supplementary material for: The hydrogen puzzle in rock-enhanced biochar: Pyrogenic coating, mineral redox and pore accessibility
Source: PLoS One. 2026 Aug 12;21(8):e0355781. doi: 10.1371/journal.pone.0355781 (PMC13465817; doi:10.1371/journal.pone.0355781)
Supplement: Supplementary file 1 [file pone.0355781.s001.pdf]

S1 Biomass and feedstock mixtures

Table S1: *Quantification of water content, carbon content and ash content of pure biomass and feedstock mixtures*

	BC450	BC450-9:1	BC450-1:1	BC450-1:9	BC750	BC750-9:1	BC750-1:1	BC750-1:9
Moisture (%)	6.5	6.1	5.7	2.5	6.5	6.4	5.1	2.8
Carbon (%)	50.7	49.1	41.7	14.5	50.7	49.7	43.0	16.7
Ash (%)	0.5	3.4	17.8	68.7	0.5	2.2	14.1	63.7

Biomass composition was analyzed by Eurofins Umwelt Ost GmbH, Bobristzsch-Hilbersdorf, Germany, according to DIN EN ISO 18122: 2016-03 and DIN EN ISO 16948: 2015-09.
