# Supplementary material for: The hydrogen puzzle in rock-enhanced biochar: Pyrogenic coating, mineral redox and pore accessibility
Source: PLoS One. 2026 Aug 12;21(8):e0355781. doi: 10.1371/journal.pone.0355781 (PMC13465817; doi:10.1371/journal.pone.0355781)
Supplement: Supplementary file 2 [file pone.0355781.s002.pdf]

S2 Rock characterization

Table S2 Physio- chemical characterization of rock powder

**Elemental composition**	**Basanite pristine (R)**	**Basanite thermally treated for 15 min at 450 °C (R450)**	**Basanite thermally treated for 15 min at 750 °C (7450)**
SiO_2 (% as oxide)	43.55	*n.a.*	*n.a.*
TiO_2 (% as oxide)	2.88	*n.a.*	*n.a.*
Al_2O_3 (% as oxide)	14.29	*n.a.*	*n.a.*
Fe_2O_3 (% as oxide)	11.15	*n.a.*	*n.a.*
MnO (% as oxide)	0.18	*n.a.*	*n.a.*
MgO (% as oxide)	8.94	*n.a.*	*n.a.*
CaO (% as oxide)	12.06	*n.a.*	*n.a.*
Na_2O (% as oxide)	2.90	*n.a.*	*n.a.*
K_2O (% as oxide)	3.48	*n.a.*	*n.a.*
P_2O_5 (% as oxide)	0.57	*n.a.*	*n.a.*
SO_3 (% as oxide)	0.05	*n.a.*	*n.a.*
C (%)	< 0.2	*n.a.*	< 0.2
N (%)	<0.05	*n.a.*	<0.05
H (%)	0.3	*n.a.*	0.4
∑AEAM-oxides (Ca, Mg, K, Na) (%)	27.38	*n.a.*	*n.a.*
Water extractable fraction of AAEMs (%)	0.30	0.24*	0.24*
Water extractable fraction of Mg (%)	0.01	0.01*	0.00*
Water extractable fraction of Ca (%)	0.01	0.02*	0.02*
Water extractable fraction of Na (%)	0.05	0.04*	0.04*
Water extractable fraction of K (%)	0.12	0.08*	0.08*
As (ppm)	<LOD	*n.a.*	*n.a.*
Pb (ppm)	<LOD	*n.a.*	*n.a.*
Cr (ppm)	195	*n.a.*	*n.a.*
Cu (ppm)	111	*n.a.*	*n.a.*
Ni (ppm)	124	*n.a.*	*n.a.*
Zn (ppm)	86	*n.a.*	*n.a.*
C-Sink Potential (t CO_2e t^{-1} rock) without ocean re-equilibration	0.451	*n.a.*	*n.a.*
C-Sink Potential (t CO_2e t^{-1} rock) with ocean re-equilibration	0.383	*n.a.*	*n.a.*
**Mineral composition: Silicates**			
Quartz SiO_2 (%)	0.5	0.6	0.6
Plagioclase $(Na,Ca)(Si,Al)_4O_8$ (%)	12.9	13.3	12.8
Clinopyroxene $(Ca,Na)(Mg,Fe,Al,Ti)(Si,Al)_2O_6$ (%)	45.6	46.1	46.5
Olivine $(Mg,Fe)_2SiO_4$ (%)	4.5	4.5	4.3
Nepheline $(Na,K)AlSiO_4$ (%)	11.3	10.7	10.8
Sodalite $Na_8Al_6Si_6O_{24}Cl_2$ (%)	0.4	0.3	0.3
Leucite $KAlSi_2O_6$ (%)	12.6	13.3	13.1
Analcime $NaAlSi_2O_6•(H_2O)$ (%)	0.8	<LOD	<LOD
Hauyne $(Na,Ca)_{4-8}Al_6Si_6(O,S)_{24}(SO_4,Cl)_{1-2}$ (%)	0.3	0.4	0.4
2:1-layer silicates $(K,H_3O)(Al,Mg,Fe)_2(Si,Al)_4O_{10}[(OH)_2,H_2O]$ (%)	2.7	2.2	2.5
Vesuvianite $Ca_{10}Mg_2Al_4(SiO_4)_5(Si_2O_7)_2(OH)_4$ (%)	1.3	1.4	1.1
**Mineral composition: Phosphates**			
Apatite $Ca_5(PO_4)_3(OH,F,Cl)$ (%)	0.9	0.9	1.0
**Mineral composition: Oxides and Hydroxides**			
Pseudobrookite $(Fe^{3+},Fe^{2+})_2(Ti,Fe^{2+})O_5$ (%)	0.2	0.5	0.6
Hematite Fe_2O_3 (%)	5.8	5.7	6.0
Ilmenite $Fe^{2+}TiO_3$ (%)	0.3	0.2	<LOD
**Physical characterisation**			
Mean* specific surface area (m^2 g^{-1})**	3.5±0.4	3.5±0.2	4.2±0.2
Grain size range (µm)	0-250	*n.a.*	*n.a.*
p80*** (µm)	62	*n.a.*	*n.a.*

** Calculated based on elemental analysis of Basanite pristine; ** mean±sd based on n=3 replicates (p>0.05); ***p80 = diameter threshold containing > 80% of total mass; grain size range and p80 adopted from Meyer zu Drewer et al. (2025). AAEMs= Alkali and alkaline earth metal. LOD= Limit of detection. N.a.= not available.*
