# Supplementary material for: The hydrogen puzzle in rock-enhanced biochar: Pyrogenic coating, mineral redox and pore accessibility
Source: PLoS One. 2026 Aug 12;21(8):e0355781. doi: 10.1371/journal.pone.0355781 (PMC13465817; doi:10.1371/journal.pone.0355781)
Supplement: Supplementary file 3 [file pone.0355781.s003.pdf]

S3 Feedstock composition second batch

Table S3: *Feedstock composition to produce rock-enhanced biochar (BC) at 450 °C (450) or 750 °C (750), containing 0% (-), 10% (9:1), 50% (1:1) or 90% (1:9) basanite rock in the rock-enhanced biochar (second production batch)*

#	Sample ID	C_{org}(%)*	Total ash (%)	Biomass ash (%)	Rock fraction (%)	Biomass fraction (%)
1	BC450	50.6	0.4	0.40	0.0	100.0
2	BC750	50.6	0.4	0.40	0.0	100.0
3	BC450-9:1	49.5	2.5	0.39	2.1	97.9
4	BC450-1:1	39.2	24.4	0.31	24.1	75.9
5	BC450-1:9	15	71.3	0.12	71.2	28.8
6	BC750-9:1	50.4	1.8	0.40	1.4	98.6
7	BC750-1:1	42	16.3	0.33	16.0	84.0
8	BC750-1:9	18.1	60.4	0.14	60.3	39.7
