# Supplementary material for: The hydrogen puzzle in rock-enhanced biochar: Pyrogenic coating, mineral redox and pore accessibility
Source: PLoS One. 2026 Aug 12;21(8):e0355781. doi: 10.1371/journal.pone.0355781 (PMC13465817; doi:10.1371/journal.pone.0355781)
Supplement: Supplementary file 4 [file pone.0355781.s004.pdf]

S4 Basic physico- chemical characterization of second batch biochars and rock-enhanced biochars

Table S4: *Basic physio- chemical properties of biochar and rock-enhanced biochar (second production batch)*

	BC450	BC450-9:1	BC450-1:1	BC450-1:9	BC750	BC750-9:1	BC750-1:1	BC750-1:9
**Mass conversion (%)**	24.2	25.4	37.4	59.5	17.6	19.4	29.9	69.0
**Rock content (%)**	0	5.7	50.2	89.1	0	8.3	49.9	89.1
**Biochar content (%)**	100	94.3	49.8	10.9	100	91.7	50.1	10.9
**Total ash (%)**	1.9	7.5	51.2	89.3	3	11	51.3	89.4
**Biochar ash (%)**	1.9	1.8	1	0.2	3	2.7	1.4	0.3
**C (%)**	87.2	81.1	46.4	9.3	94.1	85.1	42.8	10.6
**C_{org} (%)**	87.2	81.1	46.4	9.3	93.9	84.9	42.8	10.6
**C_{org_daf} (% of C_{org})**	1.1	1.1	1.1	1.2	1.0	1.0	1.1	1.0
**TIC (%)**	< 0.1	< 0.1	< 0.1	< 0.1	0.2	0.2	< 0.1	< 0.1
**Carbonate CO_2 (%)**	< 0.4	< 0.4	< 0.4	< 0.4	0.6	0.8	< 0.4	< 0.4
**H (%)**	3.1	3	1.6	0.6	1.1	0.9	0.7	0.6
**N (%)**	0.44	0.41	0.3	0.18	0.65	0.6	0.36	0.22
**S (%)**	< 0.03	< 0.03	< 0.03	< 0.03	< 0.03	< 0.03	< 0.03	< 0.03
**O (%)**	7.4	8	0.5	0.6	1.1	2.3	4.9	-0.8
**H:C_{org} molar ratio**	0.42	0.44	0.41	0.77	0.14	0.13	0.19	0.67
**H_{Bio}:C_{org} molar ratio**	0.42	0.44	0.37	0.43	0.14	0.12	0.15	0.37
**Main elements as oxides**								
**Al_2O_3 (%)**	0.03	0.92	6.96	12.77	0.08	1.30	7.03	12.16
**CaO (%)**	0.59	1.17	5.79	9.73	0.94	1.71	5.90	10.01
**Fe_2O_3 (%)**	0.05	0.80	5.94	9.82	0.11	1.20	6.05	10.91
**K_2O (%)**	0.24	0.39	1.79	2.86	0.33	0.56	1.80	2.95
**MgO (%)**	0.10	0.61	4.30	7.95	0.18	0.94	4.57	7.69
**Na_2O (%)**	0.03	0.23	1.69	2.86	0.04	0.31	1.64	2.86
**P_2O_5 (%)**	0.06	0.08	0.31	0.54	0.09	0.11	0.31	0.54
**SO_3 (%)**	0.02	0.02	*n.a.*	*n.a.*	0.02	0.02	0.05	*n.a.*
**MnO (%)**	0.07	2.93	22.07	39.38	0.11	4.21	22.26	39.43
**SiO_2 (%)**	0.18	0.08	0.15	0.18	0.30	0.11	0.15	0.18
**TiO_2 (%)**	0.01	0.17	1.38	2.41	0.01	0.25	1.39	2.41
**Trace elements***								
**Arsenic (mg kg^{-1})**	< 0.8	<0.8	1.3	1.7	< 0.8	< 0.8	1	1.9
**Lead (mg kg^{-1})**	< 2	<2	8	4	< 2	< 2	3	4
**Boron (mg kg^{-1})**	6.0	5	10		9.0	7	5	4
**Cadmium (mg kg^{-1})**	< 0.2	<0.2	4.2	0.3	< 0.2	<0.2	<0.2	0.4
**Chromium (mg kg^{-1})**	2.00	35.0	85.0	126.0	17.0	59.0	98.0	135.0
**Copper (mg kg^{-1})**	3.00	10.0	35.0	56.0	6.0	13.0	35	54
**Nickel (mg kg^{-1})**	< 1	17	61	94	7.0	25.0	71.0	93.0
**Mercury (mg kg^{-1})**	< 0.07	< 0.07	<0.07	<0.07	< 0.07	< 0.07	< 0.07	< 0.07
**Silver (mg kg^{-1})**	< 5	<5	<5	<5	< 5	< 5	< 5	< 5
**Zinc (mg kg^{-1})**	35.00	46	60.0	73.0	10.0	32.0	54.0	71.0
