# Supplementary material for: The hydrogen puzzle in rock-enhanced biochar: Pyrogenic coating, mineral redox and pore accessibility
Source: PLoS One. 2026 Aug 12;21(8):e0355781. doi: 10.1371/journal.pone.0355781 (PMC13465817; doi:10.1371/journal.pone.0355781)
Supplement: Supplementary file 5 [file pone.0355781.s005.pdf]

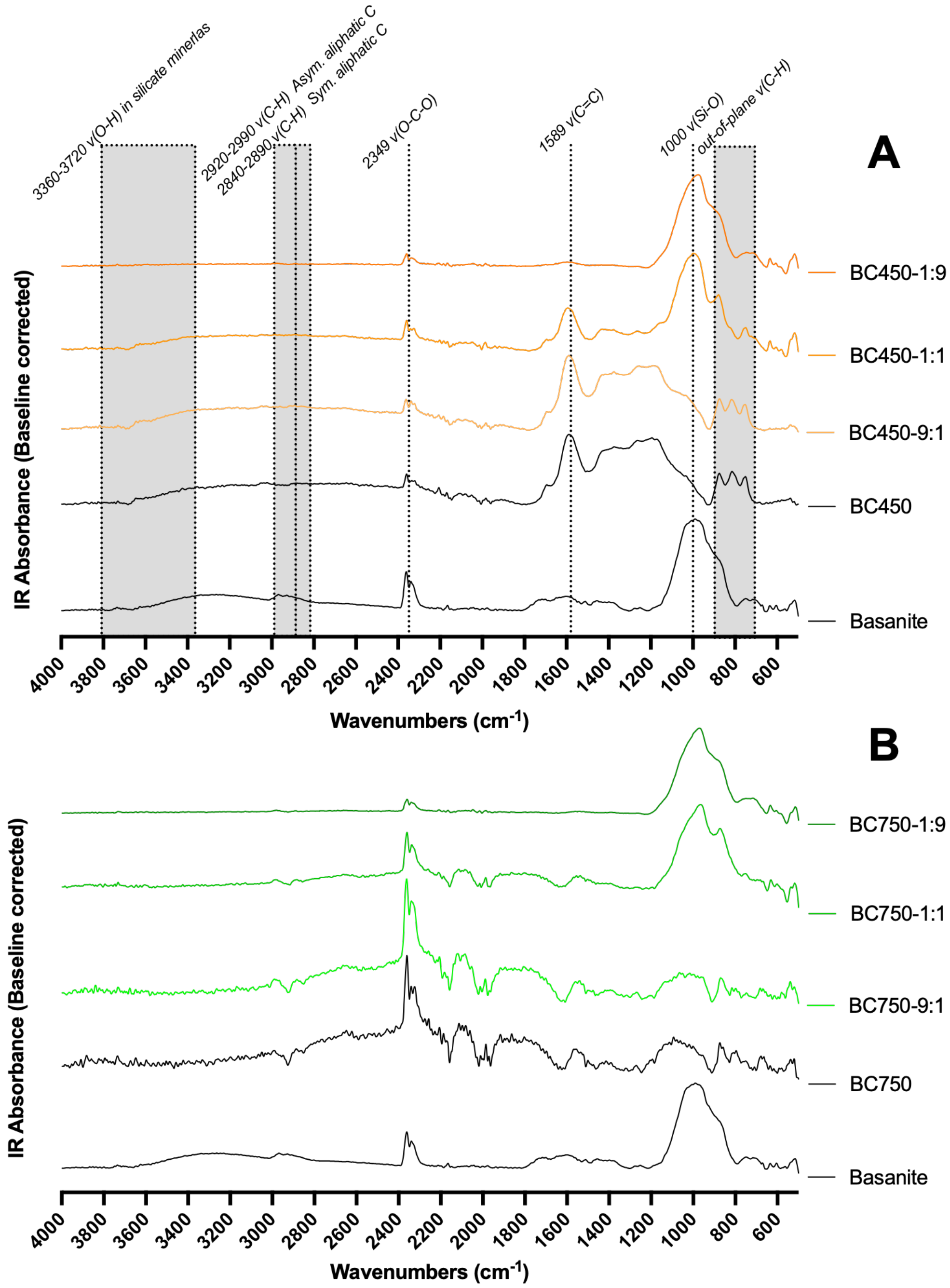

Figure S5: *Infra-red absorbance spectra obtained via Fourier transform infrared spectroscopy in attenuated total reflectance mode.* ***(A)*** *Pure biochar produced at 450 °C (BC450), and rock-enhanced biochars with 10% (-9:1), 50% (-1:1) and 90% (-1:9) rock content produced at 450 °C.* ***(B)*** *Pure biochar produced at 750 °C (BC750), and rock-enhanced biochars with 10% (-9:1), 50% (-1:1) and 90% (-1:9) rock content produced at 750 °C. Both panels include the reference spectra of pure basanite (silicate rock powder). Relevant absorption bands are highlighted in the upper panel. Note that for signals relating to carbon bonds (e.g. aromatic C=C at 1589 cm^{-1}) there is a dilution effect (i.e., reduced signal strength) for samples with higher rock content and proportionally lower carbon content. The signal at 2349 cm^{-1} originates from CO_2 in the samples pore system.*
