# Supplementary material for: The hydrogen puzzle in rock-enhanced biochar: Pyrogenic coating, mineral redox and pore accessibility
Source: PLoS One. 2026 Aug 12;21(8):e0355781. doi: 10.1371/journal.pone.0355781 (PMC13465817; doi:10.1371/journal.pone.0355781)
Supplement: Supplementary file 6 [file pone.0355781.s006.pdf]

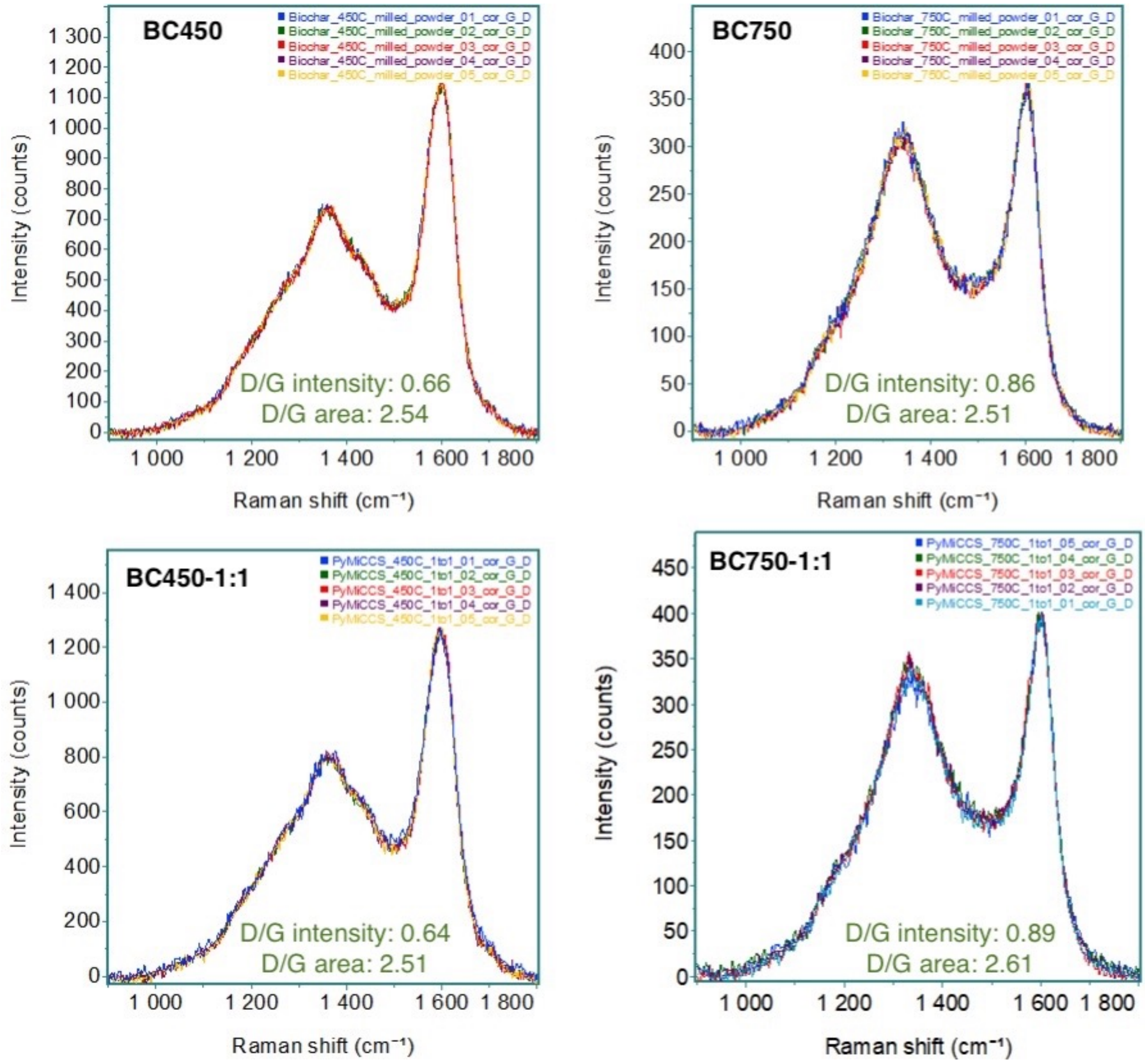

Figure S6: *Raman spectra obtained from ground samples of pure biochar produced at 450 °C (BC450), pure biochar produced at 750 °C (BC750), rock-enhanced biochar produced at 450 °C (BC450-1:1) and rock-enhanced biochar produced at 750 °C (BC750-1:1). Each panel shows the spectra obtained from n=5 scans (color coded) along with the mean D-band versus G-band intensity ratio (height of peak) and area ratio (area of peak).*
