# Supplementary material for: The hydrogen puzzle in rock-enhanced biochar: Pyrogenic coating, mineral redox and pore accessibility
Source: PLoS One. 2026 Aug 12;21(8):e0355781. doi: 10.1371/journal.pone.0355781 (PMC13465817; doi:10.1371/journal.pone.0355781)
Supplement: Supplementary file 7 [file pone.0355781.s008.pdf]

S7 Main and trace elements of biochars and rock-enhanced biochars of the first production batch

Table S7: *Main- and trace-elements in biochar and rock-enhanced biochar (BC) produced at 450 °C (450) or 750 °C (750), containing 10% (9:1), 50% (1:1) or 90% (1:9) basanite rock powder.*

	BC450	BC450-9:1	BC450-1:1	BC450-1:9	BC750	BC750-9:1	BC750-1:1	BC750-1:9
**Main elements as oxides**								
**Al_2O_3 (%)**	0.04	0.89	6.91	11.97	0.04	1.29	6.72	12.34
**CaO (%)**	0.63	0.98	5.43	9.47	0.85	1.61	5.49	9.83
**Fe_2O_3 (%)**	0.15	0.93	6.30	10.81	0.20	1.32	6.41	11.26
**K_2O (%)**	0.22	0.35	1.84	3.04	0.37	0.63	1.85	3.22
**MgO (%)**	0.12	0.57	4.15	7.59	0.16	0.88	4.16	7.69
**Na_2O (%)**	0.02	0.20	1.48	2.59	0.03	0.30	1.49	2.86
**P_2O_5 (%)**	0.06	0.07	0.31	0.45	0.08	0.11	0.31	0.54
**SO_3 (%)**	0.02	0.02	<LOQ	<LOQ	0.04	0.03	<LOQ	<LOQ
**MnO (%)**	0.08	0.06	0.15	0.18	0.11	0.10	0.15	0.18
**SiO_2 (%)**	0.17	3.09	23.19	40.81	0.29	4.40	22.93	41.93
**TiO_2 (%)**	0.00	0.17	1.38	2.41	0.01	0.25	1.33	0.18
**Trace elements**								
**Arsenic (mg kg^{-1})**	< 0.8	1.3	< 0.8	0.9	< 0.8	< 0.8	< 0.8	1
**Lead (mg kg^{-1})**	< 2	< 2	< 2	2	< 2	< 2	< 2	< 2
**Boron (mg kg^{-1})**	8	6	5	4	10	9	7	5
**Cadmium (mg kg^{-1})**	< 0.2	< 0.2	< 0.2	< 0.2	< 0.2	< 0.2	< 0.2	< 0.2
**Chromium (mg kg^{-1})**	11	41	75	51	13	34	86	57
**Copper (mg kg^{-1})**	5	13	34	50	6	10	31	50
**Nickel (mg kg^{-1})**	3	16	62	100	4	16	80	97
**Mercury (mg kg^{-1})**	< 0.07	< 0.07	< 0.07	< 0.07	< 0.07	< 0.07	< 0.07	< 0.07
**Silver (mg kg^{-1})**	< 5	< 5	< 5	< 5	< 5	< 5	< 5	< 5
**Zinc (mg kg^{-1})**	40	52	73	68	11	22	57	69

LOQ = Limit of quantification.
