# Supplementary material for: The hydrogen puzzle in rock-enhanced biochar: Pyrogenic coating, mineral redox and pore accessibility
Source: PLoS One. 2026 Aug 12;21(8):e0355781. doi: 10.1371/journal.pone.0355781 (PMC13465817; doi:10.1371/journal.pone.0355781)
Supplement: Supplementary file 8 [file pone.0355781.s009.pdf]

S8 Hydrogen in small hydrocarbons

Table S8: *Hydrogen in the rock fraction and in low molecular weight hydrocarbons*

Parameter	BC750	BC750-1:9
Corg (%)	93.9	9.6
Corg (mg g^{-1})	939	96
Corg (mol g^{-1})	0.078	0.008
H (%)	1.3	0.7
H (mg g^{-1})	13	7
H (mol g^{-1})	0.013	0.007
molar H:Corg	0.16	0.87
molar H_{Bio}:Corg*	0.16	0.53
$C_{volHAWK}$ (mg g^{-1})	0.28	0.32
H associated with $C_{volHAWK}$ (mg g^{-1})**	4.7 x10^{-5}	5.3 x10^{-5}
non-BC_{HyPy} (mg g^{-1})	25.35	2.69
H associated with non-BC_{HyPy} (mg g^{-1})***	1.3 x10^{-3}	1.4 x10^{-4}

** Calculated by equation 6, considering only carbon and hydrogen of the RE-biochars biogenic fraction (f_{Bio}). The exclusion of geogenic hydrogen explains 50% of the delta between the H:C_{org} ratios of BC750 and BC750-1:9.*

*** Assuming a dummy molar H:C_{org} ratio of 2 relating to the aliphatic carbon compounds.*

**** Assuming a dummy molar H:C_{org} ratio of 0.625 relating to the aromatic four-ring system of pyrene.*
