# Supplementary material for: The hydrogen puzzle in rock-enhanced biochar: Pyrogenic coating, mineral redox and pore accessibility
Source: PLoS One. 2026 Aug 12;21(8):e0355781. doi: 10.1371/journal.pone.0355781 (PMC13465817; doi:10.1371/journal.pone.0355781)
Supplement: Supplementary file 9 [file pone.0355781.s010.pdf]

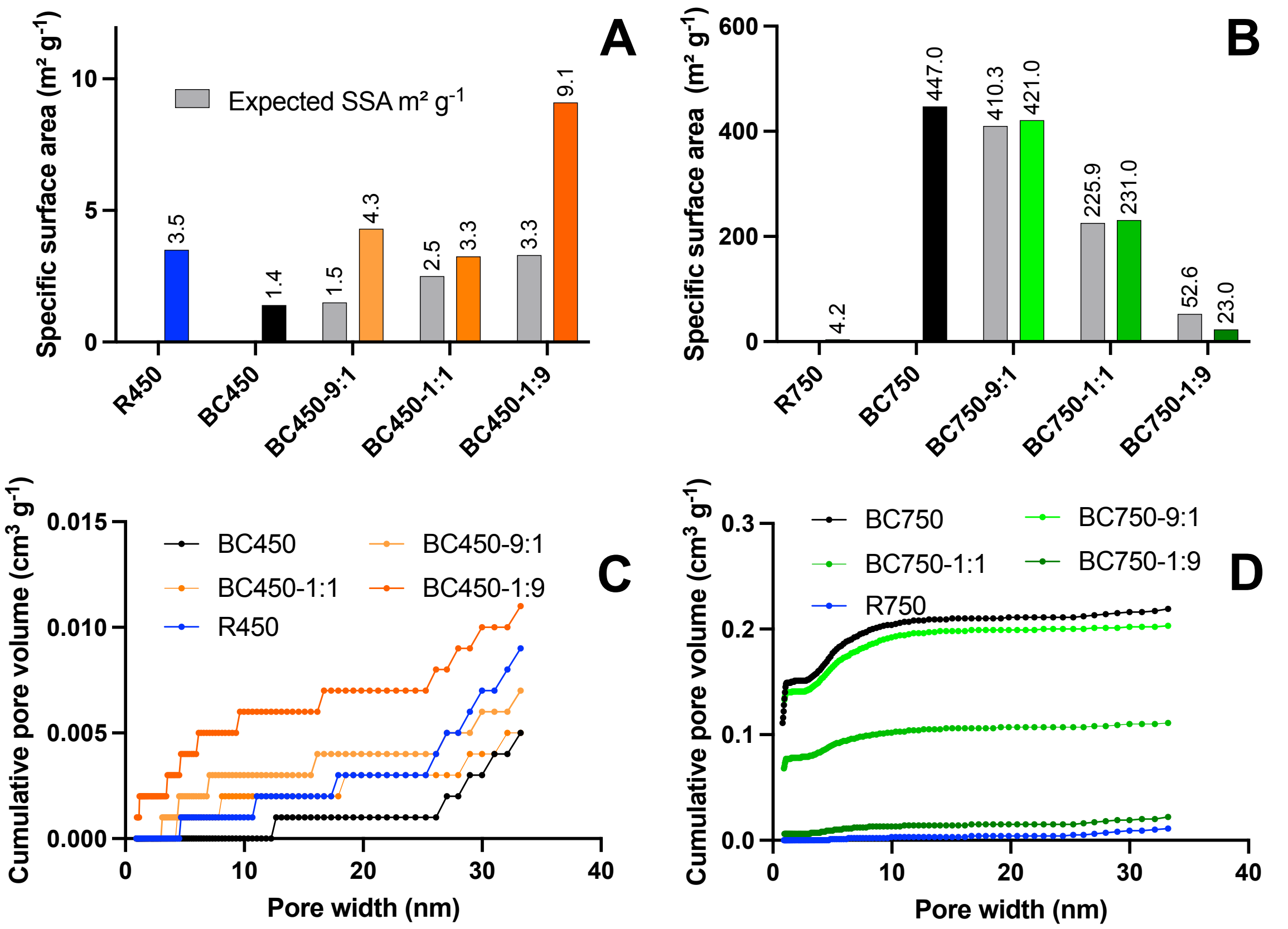

Figure S9: *Panel* ***(A+B)*** *show the specific surface area (SSA) in* m^2 g^{-1} *ad quantified by* N_2 *adsorption isotherms along with the expected SSA (grey) calculated based on the SSA of pure biochar and pure basanite under consideration of their respective mass fractions in the rock-enhanced biochars. Complementary, panel* ***(C+D)*** *show the (cumulative) pore volume distribution. R= rock (basanite), BC= biochar, 450/750 °C refer to the highest treatment temperature during pyrolysis. The rock content in rock-enhanced biochars ranges from 10% (9:1) to 90% (1:9).*
