# Supplementary material for: The hydrogen puzzle in rock-enhanced biochar: Pyrogenic coating, mineral redox and pore accessibility
Source: PLoS One. 2026 Aug 12;21(8):e0355781. doi: 10.1371/journal.pone.0355781 (PMC13465817; doi:10.1371/journal.pone.0355781)
Supplement: Supplementary file 10 [file pone.0355781.s011.pdf]

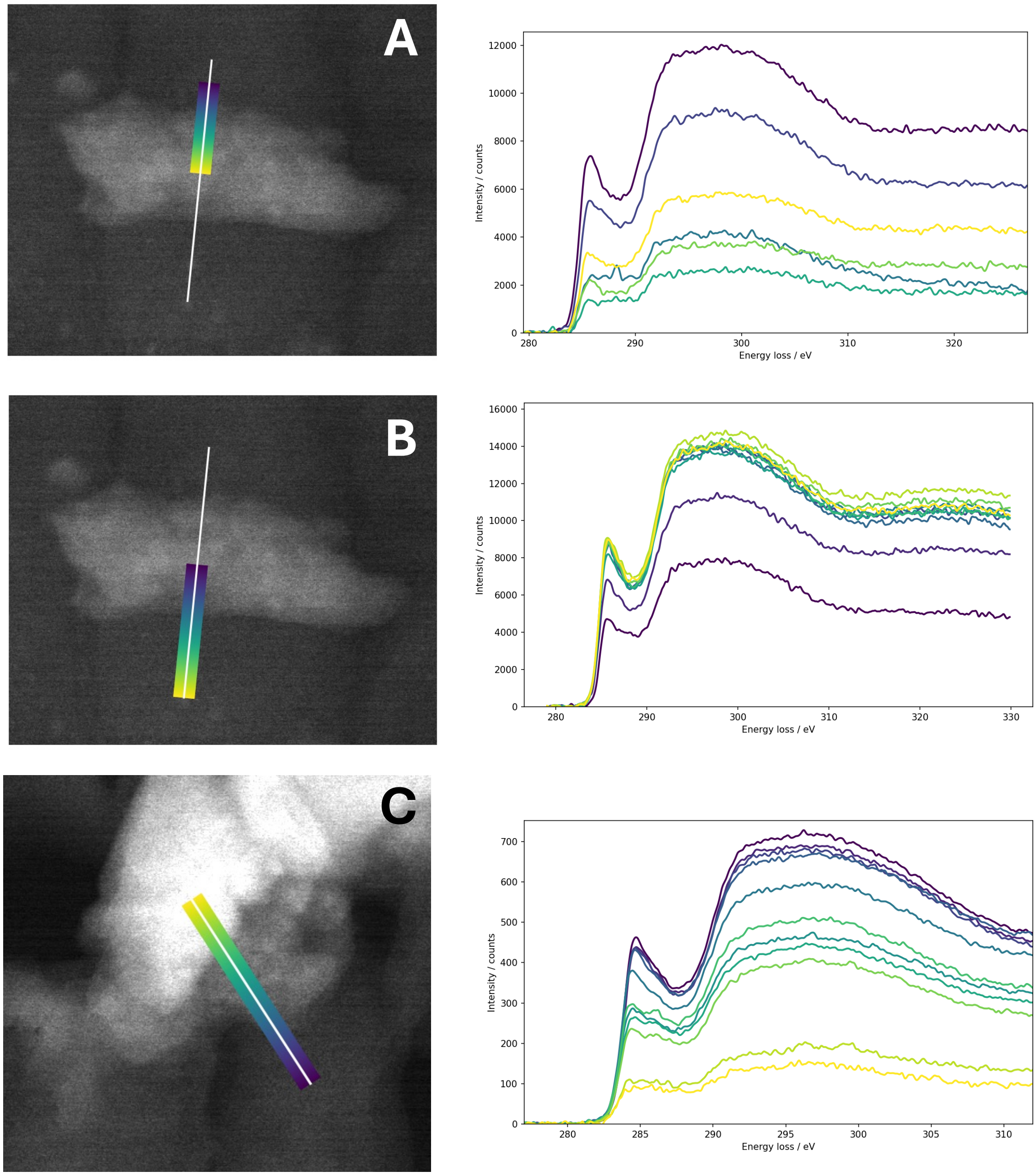

Figure S10: *Left panels **(A,B,C):** Scanning transmission electron micrographs obtained in high-angle annular dark field mode (HAADF), showing ultrathin cross section of a BC750-1:1 pellet. Mineral particles (white) are embedded in the surrounding biochar matrix (dark) with indicated measurement transect (color-coded) spanning the pure biochar (blue to purple) over the mineral-biochar interface (green to turquoise) into the rock particle (yellow to light green). Right panels: Corresponding Electron Energy Loss Spectroscopy (EELS) spectrograms obtained along the measurement transect. In panel **(B)** the beam trajectory was inverted to. Note that the Si-O peaks are cropped from the spectra.*
