# Supplementary material for: The hydrogen puzzle in rock-enhanced biochar: Pyrogenic coating, mineral redox and pore accessibility
Source: PLoS One. 2026 Aug 12;21(8):e0355781. doi: 10.1371/journal.pone.0355781 (PMC13465817; doi:10.1371/journal.pone.0355781)
Supplement: Supplementary file 11 [file pone.0355781.s012.pdf]

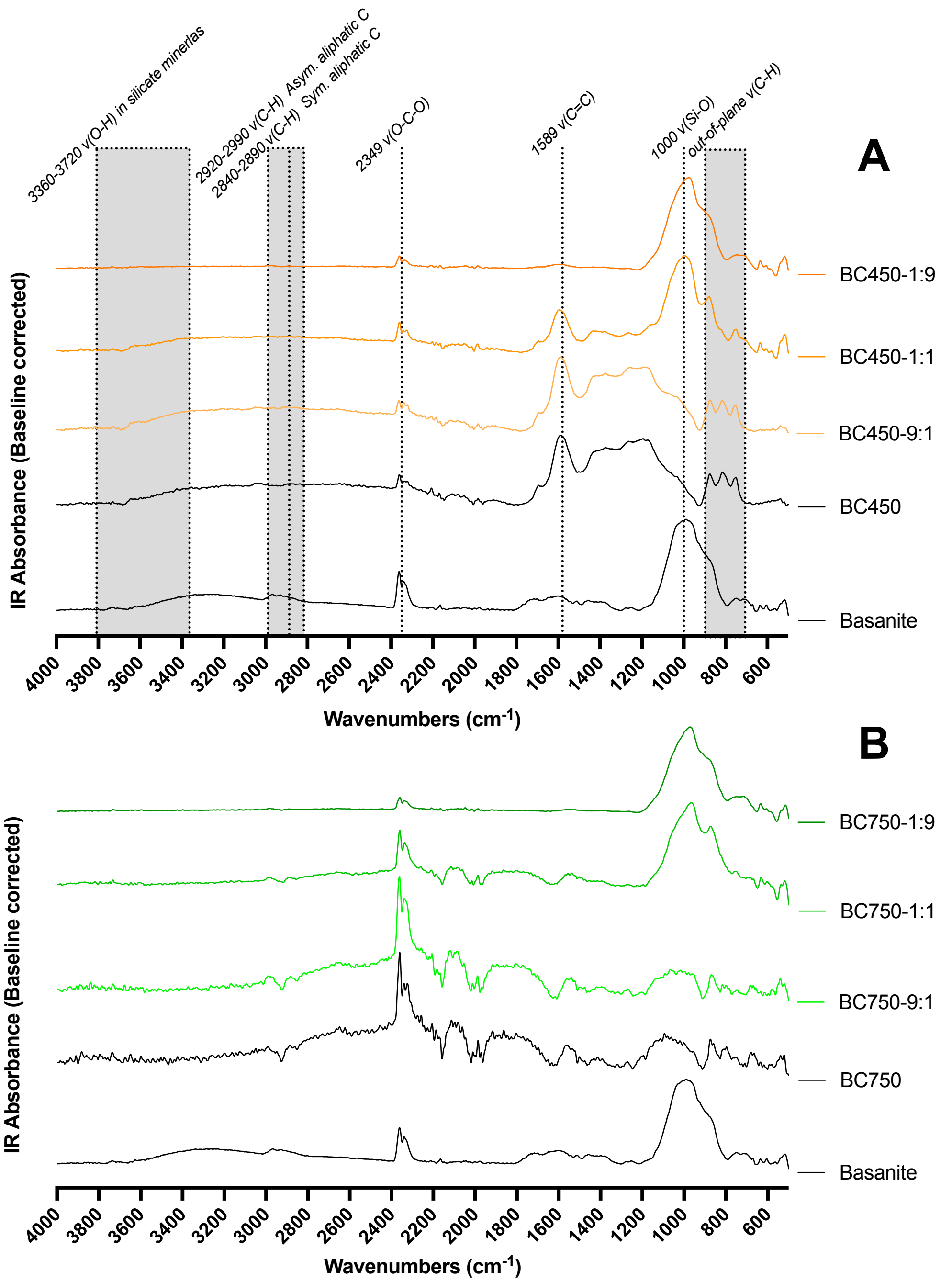

Figure S5: *Infra-red absorbance spectra obtained via Fourier transform infrared spectroscopy in attenuated total reflectance mode.* ***(A)*** *Pure biochar produced at 450 °C (BC450), and rock-enhanced biochars with 10% (-9:1), 50% (-1:1) and 90% (-1:9) rock content produced at 450 °C.* ***(B)*** *Pure biochar produced at 750 °C (BC750), and rock-enhanced biochars with 10% (-9:1), 50% (-1:1) and 90% (-1:9) rock content produced at 750 °C. Both panels include the reference spectra of pure basanite (silicate rock powder). Relevant absorption bands are highlighted in the upper panel. Note that for signals relating to carbon bonds (e.g. aromatic C=C at 1589 cm^{-1}) there is a dilution effect (i.e., reduced signal strength) for samples with higher rock content and proportionally lower carbon content. The signal at 2349 cm^{-1} originates from CO_2 in the samples pore system.*

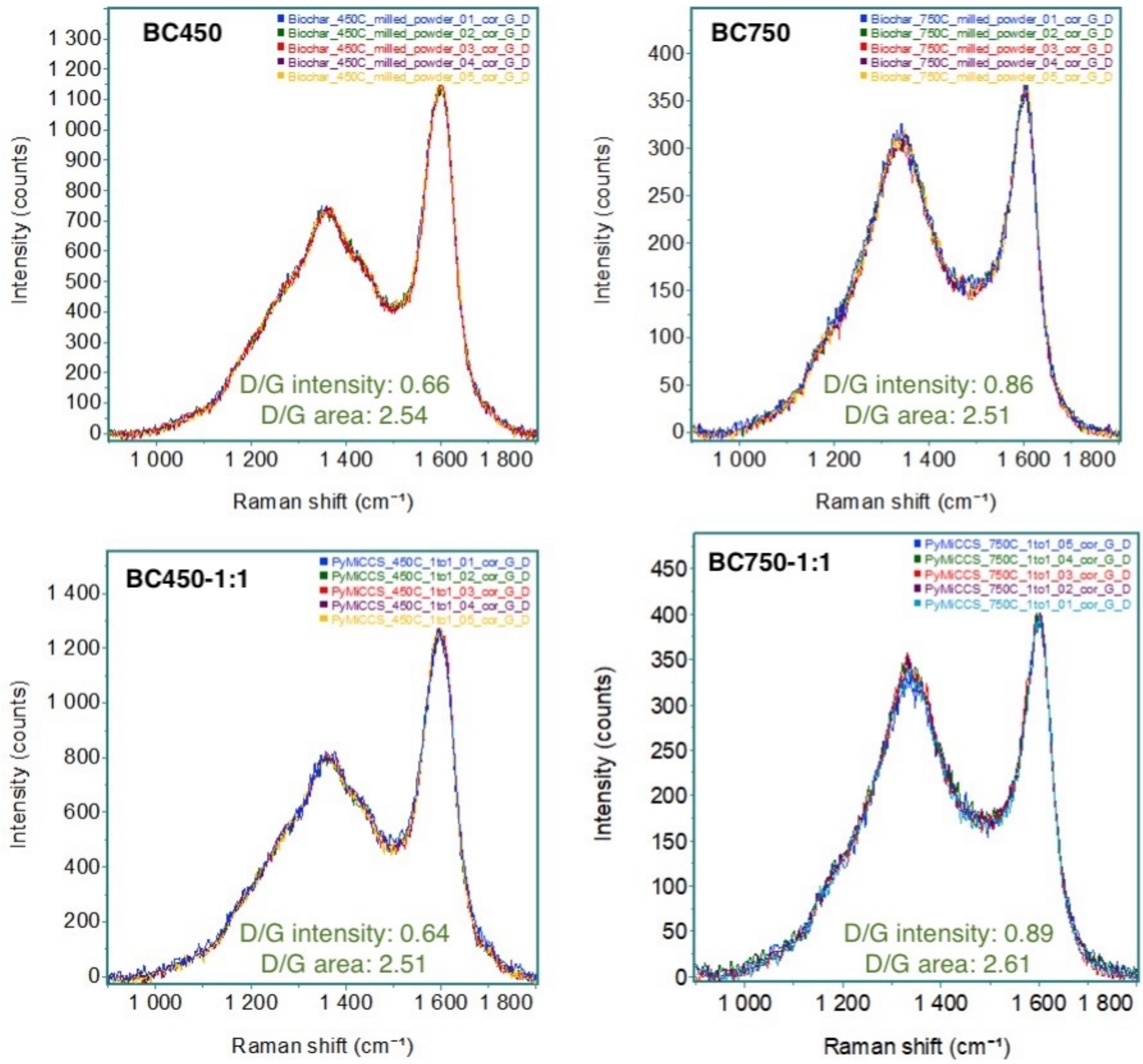

Figure S6: *Raman spectra obtained from ground samples of pure biochar produced at 450 °C (BC450), pure biochar produced at 750 °C (BC750), rock-enhanced biochar produced at 450 °C (BC450-1:1) and rock-enhanced biochar produced at 750 °C (BC750-1:1). Each panel shows the spectra obtained from n=5 scans (color coded) along with the mean D-band versus G-band intensity ratio (height of peak) and area ratio (area of peak).*

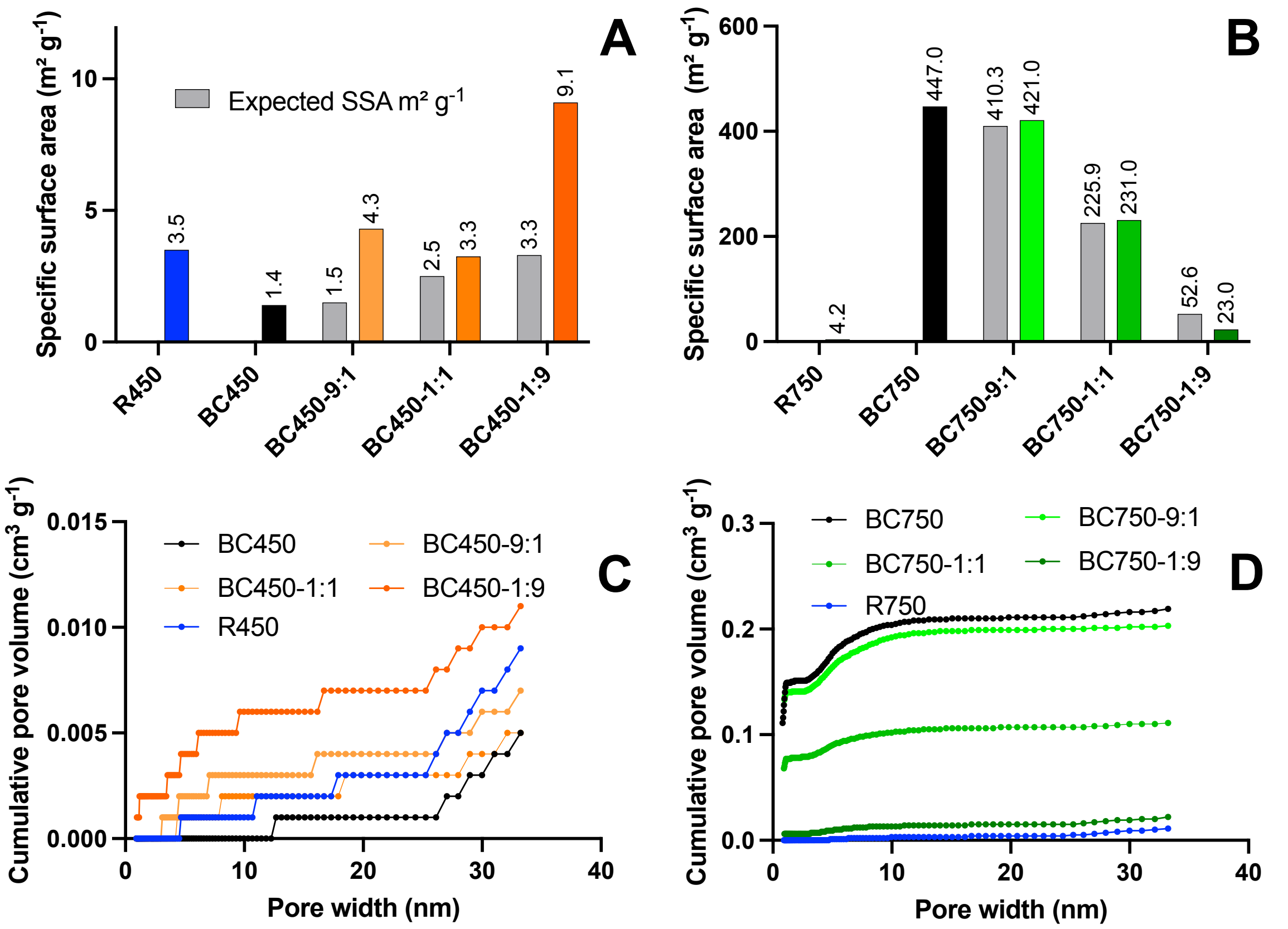

Figure S9: *Panel* ***(A+B)*** *show the specific surface area (SSA) in* m^2 g^{-1} *ad quantified by* N_2 *adsorption isotherms along with the expected SSA (grey) calculated based on the SSA of pure biochar and pure basanite under consideration of their respective mass fractions in the rock-enhanced biochars. Complementary, panel* ***(C+D)*** *show the (cumulative) pore volume distribution. R= rock (basanite), BC= biochar, 450/750 °C refer to the highest treatment temperature during pyrolysis. The rock content in rock-enhanced biochars ranges from 10% (9:1) to 90% (1:9).*

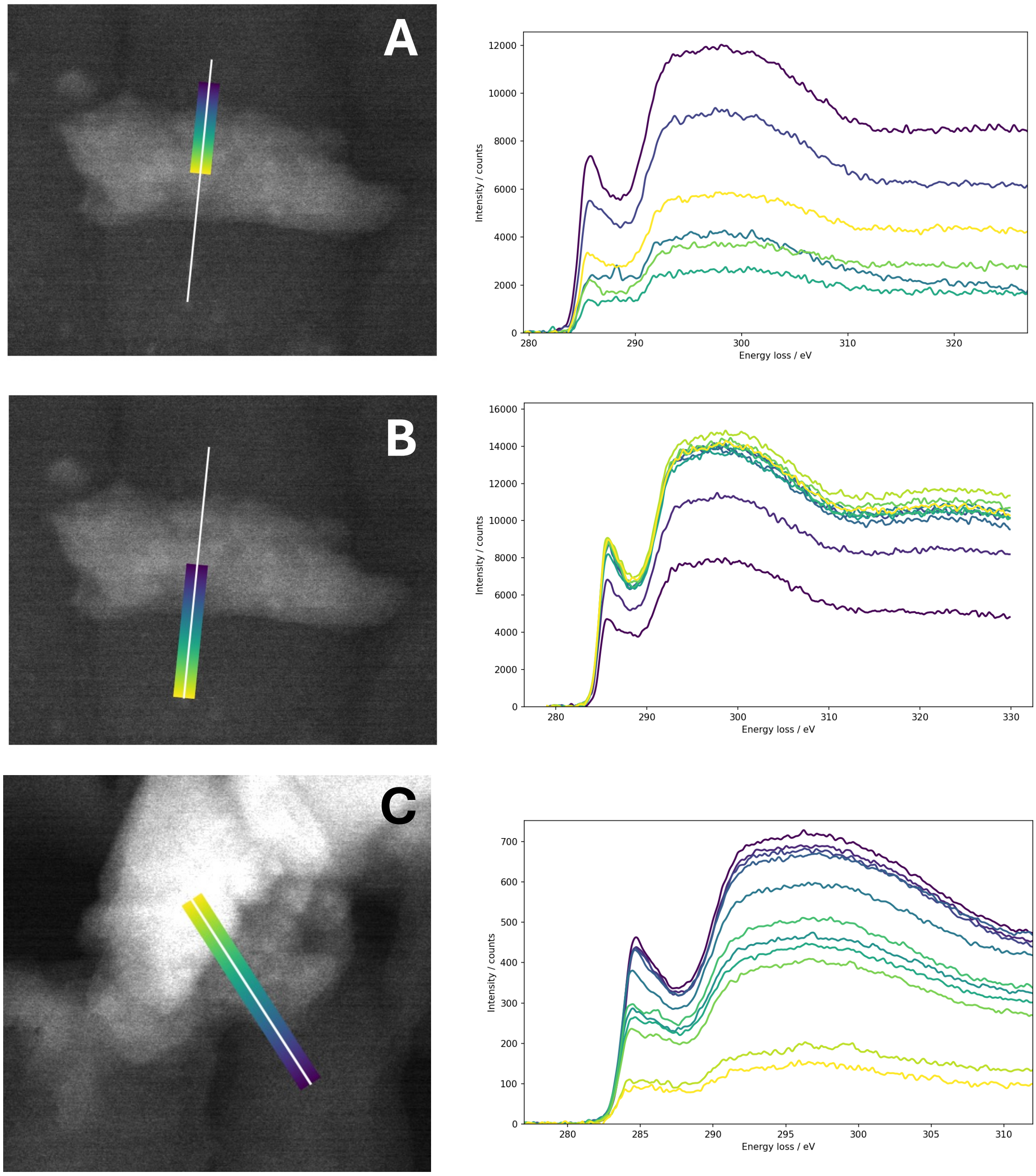

Figure S10: *Left panels* ***(A,B,C):*** *Scanning transmission electron micrographs obtained in high-angle annular dark field mode (HAADF), showing ultrathin cross section of a BC750-1:1 pellet. Mineral particles (white) are embedded in the surrounding biochar matrix (dark) with indicated measurement transect (color-coded) spanning the pure biochar (blue to purple) over the mineral-biochar interface (green to turquoise) into the rock particle (yellow to light green). Right panels: Corresponding Electron Energy Loss Spectroscopy (EELS) spectrograms obtained along the measurement transect. In panel* ***(B)*** *the beam trajectory was inverted to. Note that the Si-O peaks are cropped from the spectra.*

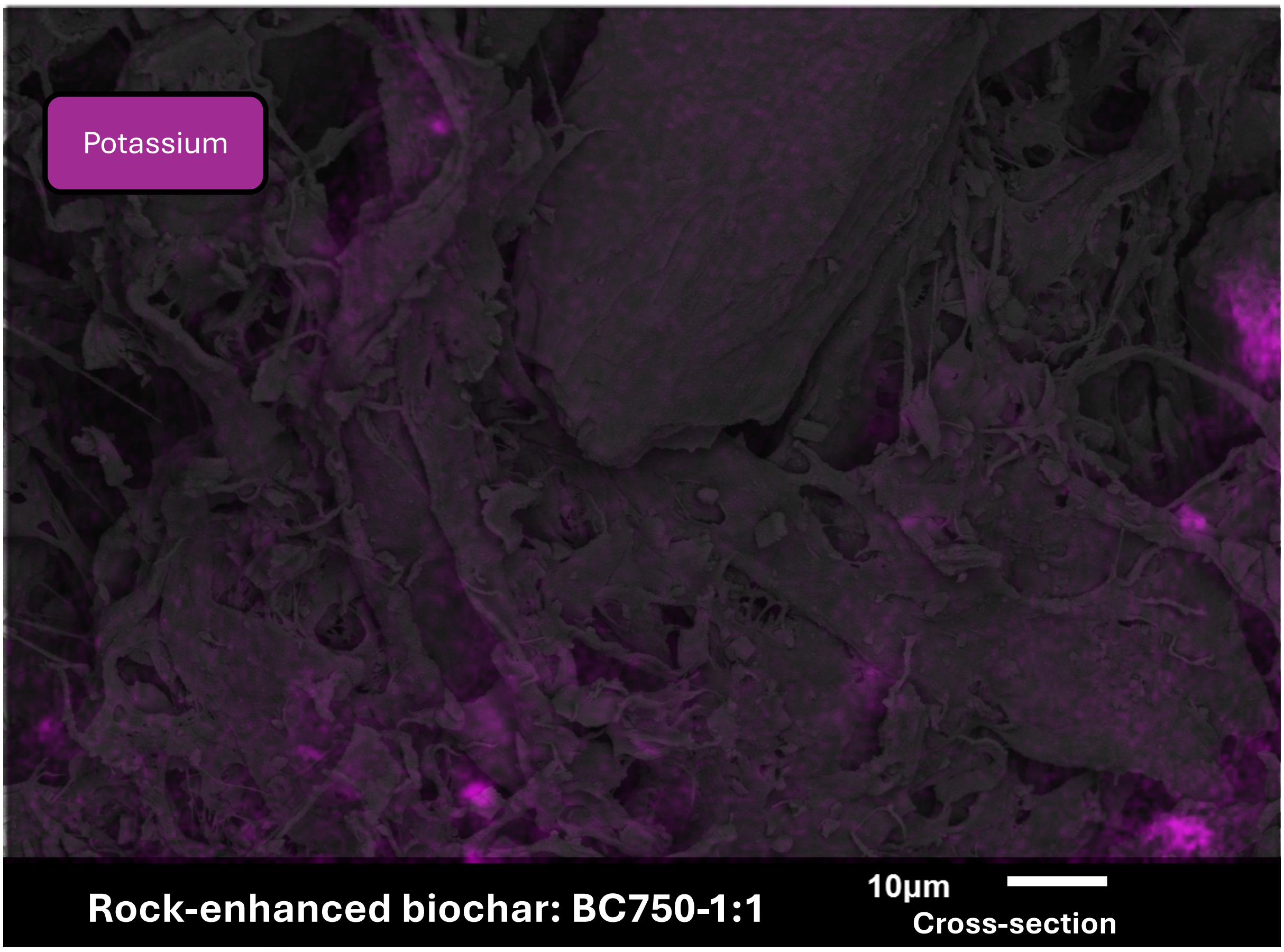

Figure S11: *Scanning electron micrograph of a cross-section of rock-enhanced biochar, produced at 750 °C, containing 50% basanite (BC750-1:1) overlayed with a mapping of superficial potassium distribution (purple) obtained via energy-dispersive X-ray spectroscopy.*
