# Supplementary material for: The hydrogen puzzle in rock-enhanced biochar: Pyrogenic coating, mineral redox and pore accessibility
Source: PLoS One. 2026 Aug 12;21(8):e0355781. doi: 10.1371/journal.pone.0355781 (PMC13465817; doi:10.1371/journal.pone.0355781)
Supplement: Supplementary file 12 [file pone.0355781.s013.pdf]

S12 M&M: The Pore Connectivity Index (PCI)

According to Lo Meo et al. (2020), the relaxation rates R_1 for porous systems can be associated to the fraction of water molecules interacting with the pore surface (f_s) by the equation:

$$R_1 = R_w + f_s \cdot (R_s - R_w) \quad (1)$$

where R_w is the relaxation rate of the bulk water (ca. 0.33 s^{-1})[31] and R_s is the intrinsic relaxation rate for the water laying on the surface of the porous material. Hence, R_1 can be related to the porosimetric parameters of the material through the following relationships:

$$R_1 = R_w + \rho \cdot (S/V) \quad (2)$$

$$R_1 = R_w + \rho \cdot (m/D) \quad (3)$$

$$R_1 = R_w + \lambda \cdot (m/D) \cdot (R_s - R_w) \quad (4)$$

where ρ is a parameter referred to as the "surface relaxivity" (in the case of clay materials or natural soils, for example, ρ values have been reported in the range of ca. 5 - 60 μ s^{-1})[32], λ represents the thickness of the hydration shell of pore walls (usually fixed as large as ca 0.3 nm) and m is a geometry parameter. Its value is a function of pore shape (m = 4 for cylindrical pores, 6 for spherical ones). Therefore, if we assume that the fast and slow components of the relaxation kinetics for wet samples adequately describe the behaviour of surface and pore water molecules, respectively, according to previous discussion, one could easily derive:

$$R_{slow} = R_w + f_s \cdot (R_{fast} - R_w) \quad (7)$$

and then:

$$f_s = (R_{slow} - R_w)/(R_{fast} - R_w) \quad (8)$$

Based on equations (3) and (4), the average pore diameters of porous materials can be related to the relaxation rates according to the following expression:

$$D = \lambda \cdot m/f_s = \lambda \cdot m \cdot (R_{fast} - R_w)/(R_{slow} - R_w) = \lambda \cdot m \cdot (1/T_{fast} - R_w)/(1/T_{slow} - R_w) \quad (9)$$

However, based on the discussion above, the relationship (9) is of difficult application because neither λ nor m values can be properly defined.

As accounted for by the relaxograms, it is possible to find a distribution of relaxation times that mirrors the parallel existence of a pore size distribution. More in detail, the normalized inverse Laplace transforms provide a frequency distribution ($P(T_1)$) for the T_1 values of the ^1H nuclei present in the sample, in such a way that an average relaxation time $<T_1>$ can be introduced as:

$$< T_1 >= \int_0^{\infty} P(T_1) T_1 dT_1 \qquad (10)$$

Hence, to apply equation (9), the average values for T_{fast} and T_{slow} can be calculated according to equation (10) by considering independently the normalized fitting expressions for the fast and the slow components instead of $P(T_1)$. Since D can be considered as a function of T_{fast} and T_{slow} according to equation (9), the normalized frequency distribution $P(T_1)$ for the isolated slow component can be transformed in a frequency distribution for D values (P'(D)) by a trivial variable exchange such as:

$$P'(D) = P\left(\left[R_w + \frac{\lambda \cdot m}{D}\left(< T_{fast} >^{-1} - R_w\right)\right]\right) \qquad (11)$$

Therefore, the average pore size can be obtained by applying equation (12):

$$< D >= \int_0^{\infty} P'(D) D dD \qquad (12)$$

However, the lack of reliable values for λ and m still forbids to define the function P'(D), thereby preventing the calculation of $<D>$ according to equation (12).

Conte and Ferro, by studying the relaxometry properties of some soils, defined two "connectivity indexes": a "Functional Connectivity Index" (FCI) and a "Structural Connectivity Index" (SCI). These parameters account for the viability of aqueous media through the channels pattern, and thus for the overall interaction abilities of the porous materials. More in detail, starting from the probability function $P(T_1)$ defined by equation (10), two arbitrary reference T_1 values (T_A and T_B) can be chosen according to the conditions:

$$\int_0^{T_A} P(T_1) dT_1 = 0.01 \text{ and } \int_0^{T_B} P(T_1) dT_1 = 0.99 \qquad (13)$$

in such a way that the interval [T_A, T_B] comprises the most significant part (98%) of the T_1 values distribution, accounting for water molecules mobility. Then, the connectivity indexes are simply defined as: FCI = T_B/T_A and SCI = $T_B - T_A$.

Aimed at applying these concepts in our context, we first reasoned to choose a slightly more restrictive significance criterion, namely keeping into account a 95% of the overall ^1H population. Thus, we defined T_A and T_B by fixing the integration values in equations (13) at 0.025 and 0.975

respectively. Then, based on the discussion of equation (9) reported above, we can define a single "Pore Connectivity Index" (PCI) as:

$$PCI = \frac{<T_{fast}> - R_w}{\left(\int_{T_A}^{T_B} P(T_{Slow}) T_{slow} dT_{slow}\right)^{-1} - R_w} \tag{14}$$

Simply speaking, PCI is related to the functional connectivity inside the porous materials. In other words, measures how strongly water inside the pores of a porous system interacts with the pore boundaries. In particular, the smaller the PCI value, the stronger is the interaction between water molecules and the walls of the pore. Conversely, as PCI value increases, the weaker become the water-pore wall interactions. Because the strength of the interactions depends on the pore size, it is possible to argue that PCI provides a quantitative evaluation of the average pore sizes of any porous material.
